# ZDHHC5 as a central regulator in a palmitoylation-associated prognostic model for lung adenocarcinoma: insights from pan-cancer and experimental analyses

**DOI:** 10.3389/fimmu.2025.1643112

**Published:** 2025-09-22

**Authors:** Sixuan Wu, Yuanbin Tang, Junfan Pan, Yaqin Zheng, Qihong Pan, Renji Liang, Haipeng Xu, Jiancheng Li

**Affiliations:** ^1^ Clinical Oncology School of Fujian Medical University, Fujian Cancer Hospital, Fuzhou, China; ^2^ Department of Oncology, The First Affiliated Hospital, Hengyang Medical School, University of South China, Hengyang, Hunan, China; ^3^ Department of Chemistry, Fuzhou University, Fuzhou, China; ^4^ Department of Thoracic Surgery, The First Affiliated Hospital, Hengyang Medical School, University of South China, Hengyang, China

**Keywords:** palmitoylation, lung adenocarcinoma, prognostic model, ZDHHC5, immune infiltration

## Abstract

**Background:**

Protein S-palmitoylation is a reversible post-translational modification that plays a significant role in tumor progression. However, the impact of palmitoylation metabolism on the prognosis and tumor microenvironment characteristics of lung adenocarcinoma (LUAD) remains unclear.

**Methods:**

Clinical and mRNA data from LUAD patients were collected from public databases. A palmitoylation-related gene cluster was constructed using consensus clustering. A prognostic model based on palmitoylation-related genes was developed using univariate Cox regression and Lasso regression analysis, and the contribution of each gene was assessed using shapley additive explanations (SHAP) analysis. The role of the key gene ZDHHC5 in LUAD was experimentally validated.

**Results:**

Cluster analysis divided patients into two groups, with group B exhibiting a better prognosis. Group A had a higher frequency of TP53 mutations, and significant differences in immune cell infiltration were observed between the two groups. A prognostic risk model, based on five key genes (ZDHHC5, ZDHHC12, ZDHHC21, LYPLA1, and PPT2), revealed significant survival differences between the high-risk and low-risk groups. Immune infiltration analysis showed differences in immune cell lineages and functional activities between risk groups. Drug sensitivity analysis indicated varying patient responses to different chemotherapy drugs across risk strata. Further analysis of ZDHHC5 expression across 33 cancers demonstrated its upregulation in multiple cancers, including LUAD. Experimental results suggest that ZDHHC5 may promote LUAD cell proliferation and metastasis both *in vivo* and *in vitro* via the PI3K/AKT pathway.

**Conclusion:**

A prognostic model based on palmitoylation-related genes offers a valuable tool for survival prediction and the development of personalized treatment strategies in LUAD. ZDHHC5, a key gene related to palmitoylation, demonstrates potential as both a therapeutic target and a prognostic marker for LUAD and other cancers.

## Introduction

Lung adenocarcinoma (LUAD), the predominant subtype of non-small cell lung cancer (NSCLC), is characterized by high aggressiveness and poor prognosis, imposing a significant burden on global healthcare systems (1–3). Despite advancements in immunotherapies and targeted therapies, overall survival (OS) rates among LUAD patients remain suboptimal, largely due to tumor heterogeneity and resistance to current treatments (4, 5). Existing diagnostic and therapeutic strategies often inadequately address the complex molecular features of LUAD, particularly the critical role of post-translational modifications, such as palmitoylation, in tumor progression and immune evasion (6, 7). These limitations highlight substantial gaps in the understanding of LUAD molecular mechanisms and underscore the urgent need for further investigation into palmitoylation-related genes as potential prognostic biomarkers and therapeutic targets. In our study, a prognostic model was developed using genes associated with palmitoylation, and their expression profiles and genomic alterations were characterized, with a specific focus on ZDHHC5. This approach aims to enhance prognostic accuracy and provide a scientific foundation for developing more effective treatment strategies.

Protein palmitoylation is a reversible lipid modification (8) occurring on certain oncogenes and tumor suppressors, and is dynamically regulated by the zinc-finger DHHC-type (ZDHHC) palmitoyltransferase family and the palmitoyl protein thioesterase family (9, 10). This modification regulates protein–protein interactions, protein stability, and signal transduction, playing a pivotal role in numerous physiological processes, as well as in tumor survival and progression (11). ZDHHC5, a key member of the palmitoyltransferase family (12), has been implicated in cancer progression and metastasis in recent studies. Aberrant expression of ZDHHC5 has been reported in multiple cancer types, including pancreatic cancer (13), esophageal cancer (14), glioma (15, 16), and lung cancer (17), and is correlated with poor prognosis. However, prognostic models based on palmitoylation-related genes remain lacking, and comprehensive systematic analyses of ZDHHC5’s role across different cancers—particularly its influence on the tumor immune microenvironment, microsatellite instability (MSI), drug sensitivity, and tumor mutational burden (TMB)—are still needed.

This study integrated the expression profiles of palmitoylation-related genes with genomic variation analysis, consensus clustering, and risk model construction to elucidate their prognostic significance in LUAD. The strength of this approach lies in linking gene expression patterns with clinical outcomes, thereby facilitating the identification of potential biomarkers for patient stratification. The primary objective was to develop a robust prognostic model based on the expression of palmitoylation-related genes that not only predicts patient survival but also uncovers molecular mechanisms underlying tumor behavior and immune regulation. By incorporating advanced statistical methods such as shapley additive explanations (SHAP) analysis, the interpretability of the model was enhanced, providing a scientific basis for personalized therapy and patient management in LUAD. Furthermore, a pan-cancer analysis of ZDHHC5 was performed alongside an in-depth investigation of its role in LUAD. Experimental results suggest that ZDHHC5 may promote LUAD cell proliferation and metastasis both *in vivo* and *in vitro* via the PI3K/AKT pathway.

## Materials and methods

### Data sources

RNA sequencing data, survival information, and clinical details were sourced from The Cancer Genome Atlas (TCGA) (https://portal.gdc.cancer.gov/) database. The GSE13213 dataset was obtained from the Gene Expression Omnibus (GEO) database (https://www.ncbi.nlm.nih.gov/geo/). A total of 31 palmitoylation-related genes were identified through a literature review and the GeneCards database (https://www.genecards.org) (18). Immunohistochemical images were sourced from the Human Protein Atlas (HPA) database. Furthermore, the activity levels of ZDHHC5 across 33 tumors were assessed using single-sample Gene Set Enrichment Analysis (ssGSEA). The flowchart illustrating the study design is shown in **Supplementary Figure S1**.

### Clustering analysis of palmitoylation-related genes

Consensus clustering methods were employed to classify samples into distinct subtypes, facilitating the exploration of potential molecular features associated with clinical outcomes. Initially, the R packages “limma”, “survival”, and “ConsensusClusterPlus” were loaded for data processing, survival analysis, and clustering analysis, respectively. For clustering, the ConsensusClusterPlus function was used to perform consensus clustering on the samples. The parameter maxK=9 allowed for the division of samples into up to nine groups, while reps=50 indicated that the clustering procedure would be repeated 50 times to ensure result stability. The k-means (km) algorithm was selected for clustering, with Euclidean distance used to measure sample similarity. During each iteration, 80% of the samples and 100% of the gene features were selected for analysis (pItem=0.8, pFeature=1) to enhance the robustness of the clustering. Ultimately, the samples were divided into two subtypes based on the clusterNum=2 parameter, and each sample’s clustering category was mapped to letter labels. This approach allows researchers to effectively categorize samples based on gene expression data, identify potential subtype features, and provide a foundation for further clinical research and exploration of molecular mechanisms.

### Construction of a prognostic model

A prognostic model was constructed using gene expression and survival data. Samples were randomly split into training and testing sets at a 1:1 ratio. LASSO Cox regression was conducted on the training set to identify prognostic genes and estimate their coefficients, with the optimal penalty parameter selected via cross-validation. Subsequently, a multivariate Cox proportional hazards model was developed based on the selected genes and refined through stepwise selection. Risk scores were calculated for each sample using the final model coefficients, and samples were stratified into high- and low-risk groups according to the median risk score of the training set. Survival differences between risk groups were evaluated using the log-rank test. Model predictive performance at specific time points was assessed by time-dependent receiver operating characteristic (ROC) curve analysis. Validation was conducted on an independent testing set, with only models meeting predefined significance and performance criteria retained.

### SHAP analysis

This study employed the SHAP analysis method, utilizing the R package “kernelshap” (version 0.9.0) to calculate the contribution of each gene in the Cox regression prognostic model, and visualized the SHAP values using the “shapviz” (version 0.10.2) package to demonstrate the influence of genes on prognostic prediction. First, single-factor significantly expressed gene expression data from the TCGA database were retrieved, and expression data and survival data files from the GEO database were merged. Next, the Cox regression prognostic model was constructed using the “survival” package (version 3.8.3) with the function coxph(Surv(futime, fustat) ~., data = rt), where futime and fustat represent follow-up time and survival status, respectively. The model was optimized using the step function for stepwise regression. The Cox regression model was built using the “glmnet” package (version 4.1.10). In SHAP analysis, the function additive_shap(multiCox, rt[,-c(1, 2)]) was used, where multiCox represents the Cox regression model, and rt[,-c(1, 2)] represents the gene expression data after removing survival time and status. Subsequently, the function shapviz(fit, X_pred = rt[,-c(1, 2)], X = rt[,-c(1, 2)], interactions = TRUE) was used to visualize the SHAP values, generating bar plots, honeycomb plots, waterfall plots, and single-sample force plots to demonstrate the influence of genes on prognostic prediction. The visualization plots were generated using the “ggplot2” package (version 3.5.2). For the risk score calculation, the risk score for each sample was computed using the training set data, and samples were classified into high-risk and low-risk groups based on trainScore > cutoff. Finally, risk classification was performed using Risk = as.vector(ifelse(trainScore > cutoff, “high”, “low”)), and the risk scores and grouping results were output to a file for subsequent analysis and clinical application.

### Prognostic modeling and ZDHHC5 survival analysis

The R package “pheatmap” was used to visualize risk scores. Risk score data were imported, sorted by risk value, and three types of plots were generated: (1) a risk score scatter plot distinguishing high- and low-risk groups; (2) a survival status plot showing patient survival time and status, with samples ordered by risk; and (3) a gene expression heatmap annotated by risk groups. A predefined color scheme was applied to differentiate risk groups and survival status, while clustering of samples and genes was disabled to preserve sample order. Kaplan–Meier (KM) curves were fitted to visualize survival distributions and display survival statistics for risk groups. Time-dependent ROC curve analysis was performed using the “timeROC” package to assess the prognostic predictive performance of risk scores and clinical variables. ROC curves for risk scores and multiple clinical features were plotted in a single figure for comparison.

Univariate Cox regression was conducted to assess the association between each variable and survival. Results were visualized using forest plots. Subsequently, multivariate Cox regression was applied to variables significant in univariate analysis to identify independent prognostic factors, and corresponding multivariate forest plots were generated. Forest plots were produced using a custom function, displaying HRs with confidence intervals, and significance was indicated by P-values. Based on the combined risk scores and clinical data, survival curves were constructed using the R packages “survival,” “regplot,” and “rms.” Samples with missing clinical values were excluded, and variables such as age were converted to numeric format. A multivariate Cox model was fitted to the survival data, and the “regplot” package was employed to generate nomograms. Calibration curves were subsequently plotted using the “rms” package, based on KM estimates and bootstrap resampling, to evaluate model predictive accuracy.

### Enrichment analysis

To investigate the functions and pathways related to ZDHHC5 in various cancers, we conducted Gene Set Enrichment Analysis (GSEA) (19) (http://www.gsea-msigdb.org/gsea/index.jsp), including Gene Ontology (GO, https://www.geneontology.org/) and Kyoto Encyclopedia of Genes and Genomes (KEGG, www.kegg.jp/kegg/kegg1.html) analyses. Additionally, the R packages **“**clusterProfiler**”**, **“**enrichplot**”**, and **“**org.Hs.eg.db**”** were employed to further annotate the pathways and functions related to ZDHHC5 (20). In the GSEA analysis, the gene list used was derived from **Supplementary Table S1**, and the criteria for determining significantly enriched gene sets were p < 0.05 and error discovery rate (FDR) < 0.05.

### Assessment of immune cell infiltration and association of ZDHHC5 with ICP genes and immunomodulators

Immune cell infiltration analysis was conducted based on CIBERSORT results. Statistically significant samples (p < 0.05) were first selected, and normal tissue samples were excluded. Immune cell proportion data were merged with risk scores and ordered accordingly. Immune function enrichment analysis was performed using ssGSEA. Gene expression data were preprocessed to remove low-expression and duplicate probes. Immune gene sets were loaded via “GSEABase,” and immune function scores were calculated and normalized using “GSVA.” After exclusion of normal samples, scores were integrated with risk data. The association between immune subtypes and risk groups was evaluated using chi-square tests. Sample names were standardized, intersecting samples identified, and rare subtypes removed. Contingency tables were constructed to assess statistical associations.

Tumor mutation burden (TMB) and risk group relationships were analyzed using merged TMB and risk score data. After selecting common samples, TMB values were log2-transformed, and risk factors were ordered. Box plots generated via “ggpubr” compared TMB across risk groups with statistical testing. Survival analysis of TMB employed the “survminer” and “survival” packages.

The TIMER2.0 database (https://cistrome.shinyapps.io/TIMER/) was used to examine the relationship between ZDHHC5 and various immune cells. Additionally, we extracted the expression data for eight common **immune checkpoint (**ICP) genes to analyze their correlation with ZDHHC5 expression. The outcomes of these analyses were visualized utilizing the **“**ggplot2**”** package in R (21). The TISIDB website (http://cis.hku.hk/TISIDB/index.php) (22) was used to produce a heatmap to depict the association between ZDHHC5 and immunomodulators in diverse cancer types.

### Relationship between ZDHHC5 and single nucleotide variant, TMB, and MSI

MSI and TMB are strongly correlated with the efficacy of immunotherapy (23, 24). To examine these relationships, we used Spearman**’**s correlation coefficient to assess the association between ZDHHC5 and both TMB and MSI. Furthermore, we explored SNV expression across various carcinomas.

### Drug sensitivity analysis

Drug sensitivity analysis was conducted based on combined risk scores and drug response data. Common samples were first identified and merged, and drug sensitivity values were log2-transformed to improve distribution. Box plots were generated for drugs showing significant differences, with colors distinguishing risk groups. Additionally, the “pRRophetic” package was employed to estimate the half-maximal inhibitory concentration (IC50) of commonly used drugs in LUAD, investigating treatment response differences among patients stratified by ZDHHC5 expression levels.

### Cell culture and transfection

BEAS-2, H1299, and HCC827 cell lines were procured from ProCell (Wuhan, China). Cells were cultured at 37 °C in a 5% CO2 atmosphere in RPMI-1640 medium supplemented with 10% fetal bovine serum (FBS). Human ZDHHC5-targeted small interfering RNAs (siRNAs) were designed by the Hanbio Co. Ltd (Shanghai, China). The siRNA sequences used were as follows: ZDHHC5si#1:5’-GAAAGAGAAGACAAUUGUAAU-3’; ZDHHC5si#2:5’-CGACACCUACCAUGUACAAGU-3’; ZDHHC5si#3:5’-CCUCAGAUGAUUCAAAGAGAU-3’; and sicontrol: 5’-UUCUCCGAACGUGUCACGUTT-3.’ The control plasmid (vector) and the ZDHHC5 overexpression plasmid (OE-ZDHHC5) were cloned into the pcDNA 3.1 (+) vector (Hanbio Co. Ltd, Shanghai, China), and HCC827 cells were transiently transfected using Lipofectamine 3000 (Invitrogen, Waltham, Massachusetts, USA) according to the manufacturer’s instructions. The cells were collected 48–72 hours after transfection.

### Real-time quantitative reverse transcription polymerase chain reaction

Total RNA was extracted using the TRIzol reagent (Invitrogen, USA), and complementary DNA was synthesized using the PrimeScript RT kit (Takara). qRT-PCR was performed using Takara SYBR Green assay. The qRT-PCR data were analyzed using the 2-ΔΔCt method, with β-actin serving as the internal control. The specific primers used were: ZDHHC5-F: 5**’**-AGACCACCTACAGCAAATCCA-3**’**; ZDHHC5-R: 5**’**-CCTGACACCTTCTTGACTCCT-3**’**; β-actin-F: 5**’**-GAGAAAATCTGGCACCACACC-3**’**; and β-actin-R: 5**’**-GGATAGCACAGCCTGGATAGCAA-3**’**.

### Western blot

Intracellular proteins were extracted using RIPA lysis buffer supplemented with 1% protease inhibitor (P1005, Beyotime, China) and phosphatase inhibitor (P1081, Beyotime, China). The concentration of the isolated proteins was measured using the bicinchoninic acid (BCA) assay (Beyotime Biotechnology). The total cell lysates were subsequently subjected to 4%–20% sodium dodecyl sulfate-polyacrylamide gel electrophoresis (SDS-PAGE), followed by transfer to polyvinylidene fluoride (PVDF) membranes. To prevent nonspecific binding, the membranes were incubated with 5% skim milk in TBST buffer for one hour and were then incubated overnight at 4 °C with primary antibodies at their recommended concentrations. After this incubation period, the membranes were exposed to horseradish peroxidase (HRP)-conjugated secondary antibodies for one hour. The visualization of protein bands was accomplished using a chemiluminescent HRP substrate in conjunction with an imaging system (Chemidoc, Bio-Rad). The primary antibodies utilized in this study, at their designated dilutions, included ZDHHC5 (Cat No: YN6038, 1:1000; ImmunoWay Biotechnology, Plano, TX, USA), PI3K(Cat No: YW8045, 1:1000; ImmunoWay Biotechnology, Plano, TX, USA), p-PI3K(Cat No: YP0765, 1:1000; ImmunoWay Biotechnology, Plano, TX, USA), AKT (Cat No: YM8463, 1:1000; ImmunoWay Biotechnology, Plano, TX, USA), p-AKT (Cat No: YM8304, 1:1000; ImmunoWay Biotechnology, Plano, TX, USA), and α-tubulin (Cat No: 11224-1-AP, 1:1000; Proteintech, Wuhan, China).

### Immunohistochemical assay

IHC staining was performed on mouse tumor samples. Sections were incubated overnight with primary antibodies against ZDHHC5 and Ki67, with sheep serum used as a negative control. Following incubation, the sections were treated with an anti-rabbit secondary antibody and a streptavidin-peroxidase complex. After staining, the sections were counterstained with hematoxylin, followed by dehydration and mounting. Staining intensity was graded as 0 (no staining), 1+ (weak), 2+ (moderate), or 3+ (strong). H-scores were calculated as the product of intensity and extent scores, independently assessed by two pathologists. The antibodies used in this study and their specified dilutions were: ZDHHC5 (Cat No: 84803-4-RR, 1:500; Proteintech, Wuhan, China) and Ki67 (Cat No: YM8189, 1:400; ImmunoWay Biotechnology, Plano, TX, USA).

### Cell proliferation and colony formation assays

To assess cell growth, 1 × 10^4 cells were transferred to each well of a 24-well plate and cultured in 10% FBS RPMI 1640 medium. The cells were collected at 24-, 48-, and 72-hour intervals, and their absorbance was measured using Cell Counting Kit (CCK)-8. Cells (500 cells/well) were transferred to 6-well plates and cultured for 10 days. The cells were fixed with formaldehyde and stained with crystal violet (Sigma-Aldrich). Cells were counted using an inverted microscope, and images were captured. Each experiment was performed in triplicate.

### Wound-healing assay

The cells were first transferred to 6-well plates at a density of 1 × 10^5 cells per well. Subsequently, a consistent wound was created by gently scratching the cell monolayer using the tip of a 10 uL plastic pipette. Cell migration was monitored at 0 h and 24 h. Each experiment was performed in triplicate.

### Transwell assays

To perform the migration assay, 2 × 10^4^ cells were suspended in 200 μL of serum-free medium and seeded into the upper chamber of a Transwell system (BD Biosciences, USA). For the invasion assay, 50 μL of Matrigel, diluted 1:8, was added to each well and incubated at 37 °C for 4 hours to allow the Matrigel to solidify. Subsequently, 5 × 10^4^ cells were suspended in 200 μL of serum-free medium and added to the upper chamber containing Matrigel. The lower chamber was filled with 600 μL of medium containing 10% fetal bovine serum (FBS) to provide the necessary nutrients and conditions for cell growth. The cells were incubated for 24 hours to complete the migration and invasion process. After incubation, the cells in the lower chamber were fixed with formaldehyde to preserve their morphology. The cells were then stained with crystal violet. To ensure accurate results, five random fields of view were selected for photography and analysis. This experiment was repeated three times to confirm the reliability and reproducibility of the findings. Through this procedure, the migration and invasion capabilities of the cells were assessed, providing valuable data for further research.

### Tumor xenografts models

The animal experimentation protocol for this study was approved by the Fujian Anburi Biological Experimental Animal Ethics Committee (IACUC FJABR2025061001). BALB/c nude mice (4–6 weeks old) were purchased from Jiangsu Jinpu Biotechnology Co., Ltd. All mice were housed on standard rodent chow in a specific pathogen-free environment at 23 °C with a 12-hour light-dark cycle. After randomization, mice were subcutaneously injected with HCC827 cells transfected with ZDHHC5 knockdown. Each group consisted of six mice, with 100 µl of a solution containing 5 × 10^6 cells injected per mouse. Tumor growth was monitored weekly by measuring the maximum (A) and minimum (B) tumor diameters, and tumor volume was calculated using the formula: volume = 0.5 × A × B. The experiment was conducted over 4 weeks. Mice were euthanized when the tumor diameter reached 1.5 cm or at the end of the experiment, and tumor tissues were collected for IHC staining to assess tumor growth and changes in related molecular markers.

### Statistical analysis

The data were assessed and visualized using R software (version 4.4.2) (https://cran.r-project.org/). GraphPad Prism 9.0 was utilized for both the visualization and statistical evaluation of the experimental data. A p < 0.05 was regarded as statistically significant.

## Results

### Expression and genomic variation analysis of palmitoylation-related genes

**Figure 1:**
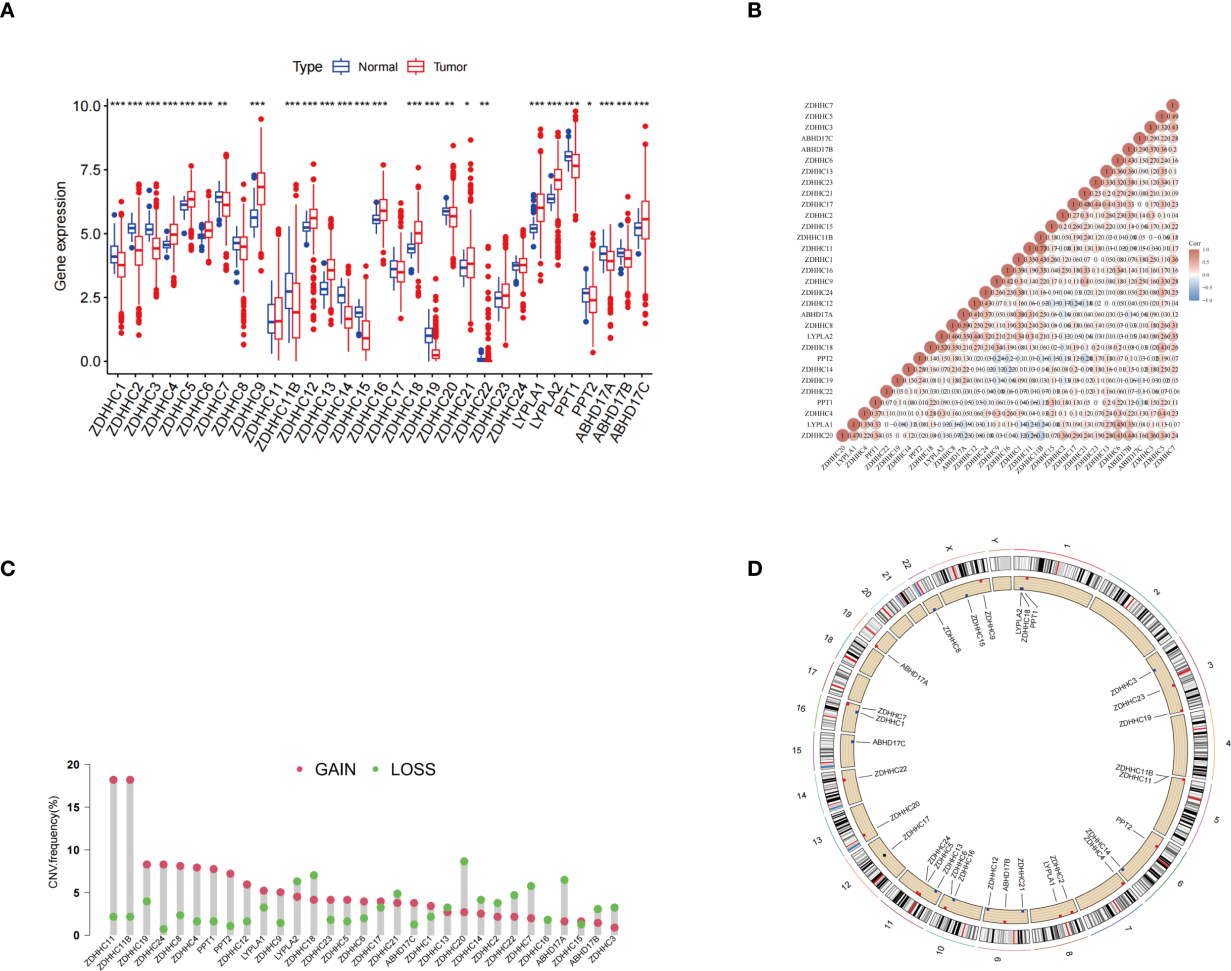
Expression and genomic variation analysis of palmitoylation-related genes. **(A)** Expression levels of palmitoylation-related genes were compared between normal tissues and LUAD tissues. **(B)** Correlation analysis of the expression patterns among palmitoylation-related genes. **(C)** Frequencies of CNVs in palmitoylation-related genes. **(D)** Schematic representation of the chromosomal distribution of palmitoylation-related genes and their corresponding CNV locations. ^*^p < 0.05; ^**^p < 0.01; ^***^p < 0.001.

**Figure 1A** illustrates the differential expression of 31 palmitoylation-related genes between normal and LUAD tumor tissues, revealing that 13 genes were downregulated while another 13 were upregulated in LUAD. A correlation heatmap of these genes is presented in **Figure 1B**. Additionally, **Figures 1C** and **D** depict the frequencies of copy number variations (CNVs) in palmitoylation-related genes and their genomic distribution, respectively. These analyses provided a deeper understanding of the expression characteristics of palmitoleic acid-related genes in LUAD and their potential biological significance, offering valuable insights for further mechanistic studies.

### Consensus clustering analysis and somatic mutation, and immune-related differences between different groups

**Figure 2:**
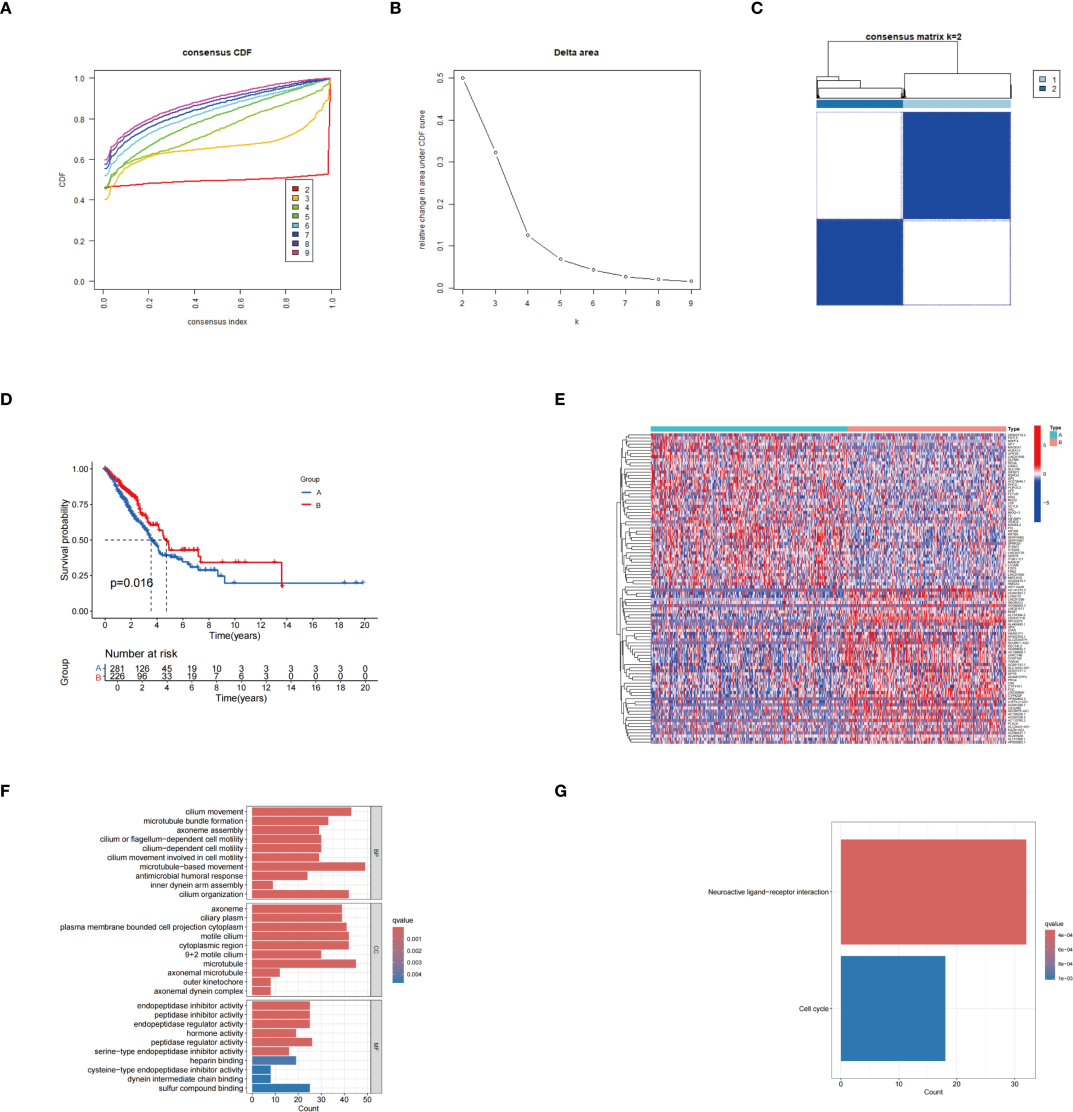
Results of consensus clustering analysis. **(A)** Consensus cumulative distribution function (CDF) curves for different cluster numbers. **(B)** Relative changes in the area under the CDF curves at varying k values. **(C)** Heatmap of the consensus matrix at k = 2, illustrating the stability of sample clustering. **(D)** KM survival curves showing the comparison of survival outcomes between groups A and B. **(E)** Heatmap depicting gene expression differences between the two identified subtypes.. **(F)** GO enrichment analysis. **(G)** KEGG enrichment analysis.

The unsupervised clustering analysis of LUAD patients was conducted based on the expression levels of 31 palmitoylation-related genes. The clustering heatmap at k=2 reveals highly consistent dark squares within the two subtypes, with clear boundaries, indicating that the clustering results are stable and reliable (**Figures 2A-C**; **Supplementary Figure S2A**). Among these clusters, group B demonstrated a better prognosis (**Figure 2D**). Heatmaps displayed the differentially expressed genes between the two clusters (**Figure 2E**). GO enrichment analysis revealed that the biological process terms included microtubule basal movement, cilium movement, and cilium organization. In terms of cellular components, enriched terms included microtubules, motile cilia, and the cytoplasmic region. The molecular function terms primarily included peptidase regulatory activity, endopeptidase inhibitory activity, and peptidase inhibitory activity (**Figure 2F**). KEGG enrichment analysis indicated that the differentially expressed genes (DEGs) were predominantly enriched in the neuroactive ligand-receptor interaction pathway (**Figure 2G**). Through these analyses, a deeper understanding of the functions of palmitoleic acid-related genes in LUAD and their potential biological significance has been gained, offering new directions for future mechanistic studies.

The distribution of somatic mutations was analyzed between group A and group B. In group B, 193 out of 219 samples (88.13%) exhibited gene mutations, whereas in group A, 257 out of 277 samples (92.78%) showed mutations. Notably, group A exhibited a significantly higher frequency of TP53 mutations (**Supplementary Figures S2B, C**). In group B, higher levels of CD8 T cells, monocytes, CD4 memory resting T cells, M0 macrophages, resting dendritic cells, and resting mast cells were observed, while in group A, elevated levels of naïve B cells, CD4 memory activated T cells, and M1 macrophages were found (**Supplementary Figure S2D**). Differential expression analysis of HLA genes revealed that HLA-DMA, HLA-DQB2, HLA-DQB1, HLA-DRB1, HLA-DPB1, HLA-DRB5, and HLA-J gene expression was upregulated in group B, whereas HLA-A and HLA-C gene expression was upregulated in group A (**Supplementary Figure S2E**). These findings offer valuable insights into the differences in the tumor immune microenvironment and may serve as a foundation for the development of personalized immunotherapy strategies.

### Construction of a risk model, SHAP analysis, and evaluation of its prognostic performance

**Figure 3:**
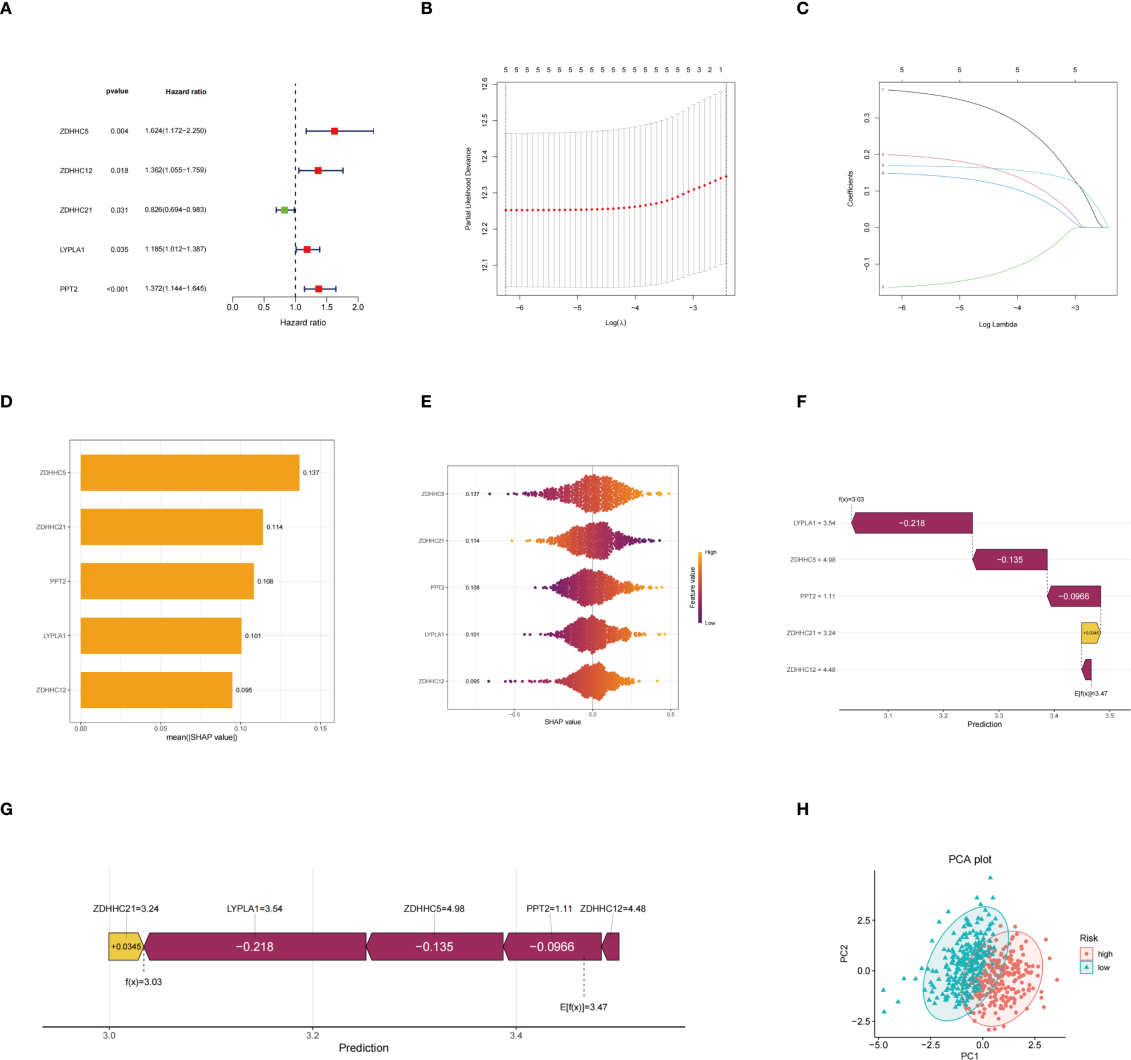
Construction of the risk model. **(A)** Hazard ratio forest plot; **(B)** Partial likelihood deviance and its standard error based on different lambda values in the LASSO regression model for selecting the optimal model parameters; **(C)** Path diagram of LASSO regression coefficients showing the changes in the regression coefficients of five genes across different lambda values; **(D)** Histogram of average SHAP values of genes indicating the relative importance of each gene’s contribution to model prediction; **(E)** SHAP value swarm plot demonstrating the distribution of SHAP values for each gene across different samples and the effect of expression levels on model prediction; **(F)** SHAP waterfall plot illustrating the positive and negative contributions of each gene to the prediction result in a representative patient sample; **(G)** SHAP force plot showing the cumulative effect from the baseline prediction value to the final prediction outcome; **(H)** PCA plot.

Univariate Cox regression analysis was conducted to identify palmitoylation-related genes significantly associated with prognosis (**Figure 3A**). Subsequently, Lasso regression analysis was conducted on these prognosis-related genes, resulting in the selection of five genes for the development of the prognostic model (**Figures 3B, C**). Risk scores were calculated using the formula provided below: Risk score = (0.3869 × ZDHHC5 expression) + (0.2069 × ZDHHC12 expression) + (–0.1710 × ZDHHC21 expression) + (0.1539 × LYPLA1 expression) + (0.1709 × PPT2 expression). The average importance of these five genes, based on SHAP values, was illustrated by bar graphs, with ZDHHC5 exhibiting the highest contribution, followed by ZDHHC21 and PPT2, indicating their prominent roles in model prediction (**Figure 3D**). The swarm plots depicted the relationship between gene expression levels and SHAP values; colors ranging from violet to orange represented low to high expression, respectively. Distinct distributions of SHAP values corresponding to high expression levels of different genes further demonstrated the impact of gene expression on risk prediction (**Figure 3E**). Waterfall plots and force diagrams for individual samples illustrated the specific contributions of genes to prediction outcomes, where genes with negative values decreased the risk prediction score, and those with positive values increased it, thereby providing an intuitive representation of the model’s internal decision-making process (**Figures 3F, G**). Finally, the PCA scatter plot clearly separated the high-risk group from the low-risk group, indicating the strong discriminatory capacity of the model features in sample risk classification (**Figure 3H**).

**Figure 4:**
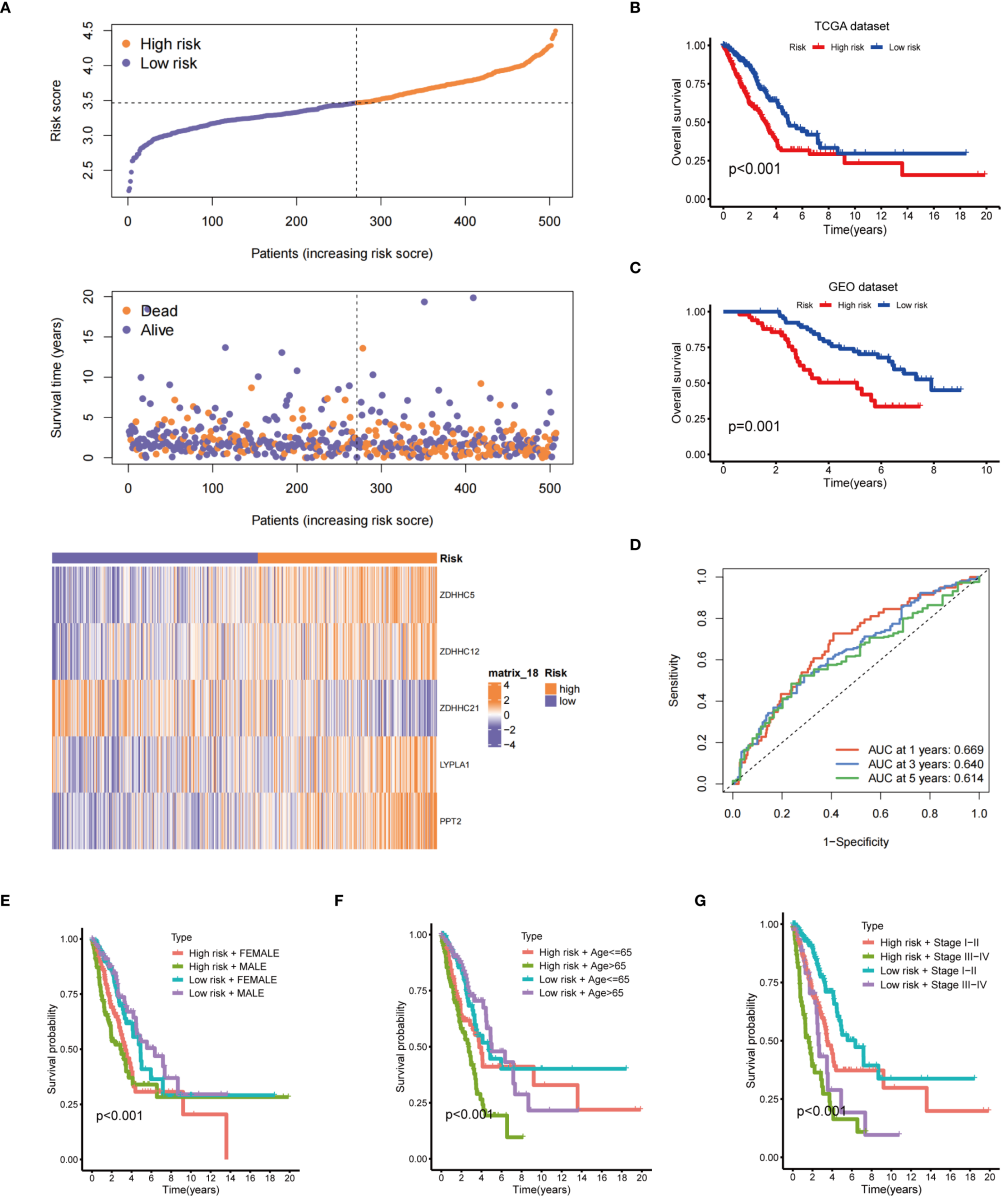
Prognostic evaluation of the risk model. **(A)** The upper panel presents the risk score distribution; the middle panel illustrates the distribution of survival status and survival time; the lower panel displays a heatmap of the expression levels of five genes associated with the risk score; **(B)** Kaplan-Meier OS curve for the TCGA dataset between the two risk groups; **(C)** Kaplan-Meier OS curve for the GEO dataset between the two risk groups; **(D)** Time-dependent ROC curves; Survival analysis stratified by gender **(E)**, age **(F)**, and clinical stage **(G)**.

The risk distribution graph demonstrated a stepwise increase in patients’ risk scores, with a marked reduction in survival time in the high-risk group (**Figure 4A**). The Kaplan-Meier analysis demonstrates that the survival rate in the low-risk group is significantly higher than that in the high-risk group (**Figure 4B**). Moreover, validation with the GEO database yielded consistent results (**Figure 4C**). The predictive performance of the risk model, assessed by the ROC curve, yielded area under the curve (AUC) values of 0.669, 0.640, and 0.614 at 1, 3, and 5 years, respectively (**Figure 4D**). Stratified survival analyses according to gender (**Figure 4E**), age (**Figure 4F**), and tumor stage (**Figure 4G**) indicated that risk scores had a significant impact on survival across all strata, with the low-risk group consistently exhibiting a more favorable prognosis. Based on the aforementioned results, the risk scoring model not only predicts OS but also offers valuable insights for developing personalized treatment strategies for patients in clinical settings, highlighting its significant clinical applicability.

**Figure 5:**
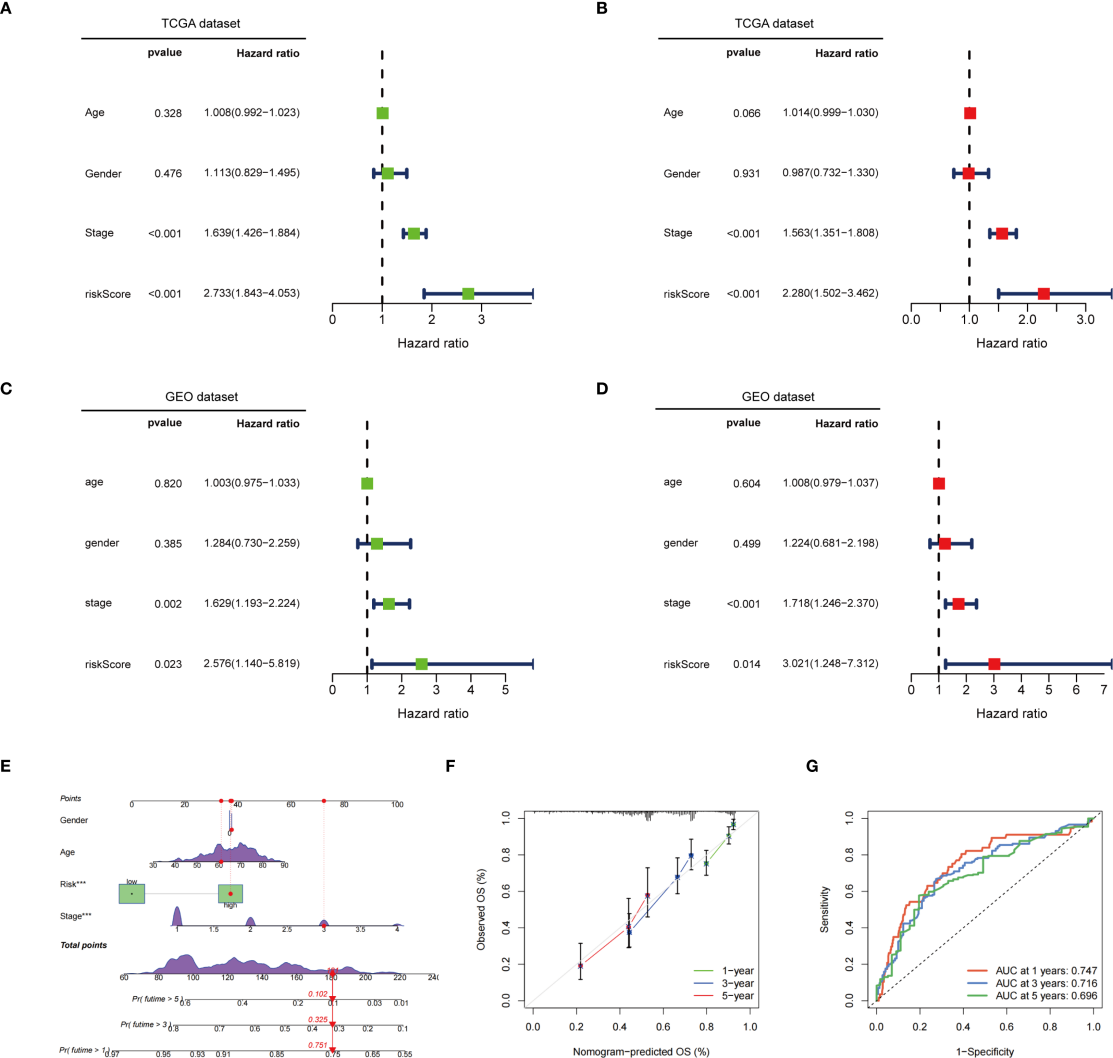
Independent prognostic value and assessment of predictive efficacy of risk models. **(A)** Univariate Cox regression analysis in the TCGA dataset; **(B)** Multivariate Cox regression analysis in the TCGA dataset; **(C)** Univariate Cox regression analysis in the GEO dataset; **(D)** Multivariate Cox regression analysis in the GEO dataset; **(E)** In the TCGA dataset, a nomogram constructed based on age, gender, risk score, and staging; **(F)** Calibration curve of the nomogram; **(G)** ROC curve of the nomogram. ^***^p < 0.001.

Univariate and multivariate Cox regression analyses demonstrated that both tumor stage and risk score significantly influenced patient survival risk, with the risk score exhibiting the highest hazard ratio, thereby emphasizing its role as a key prognostic factor (**Figures 5A, B**). Furthermore, validation using the GEO database confirmed that the risk score serves as an independent prognostic factor in both univariate and multivariate Cox regression analyses (**Figures 5C, D**). A nomogram integrating sex, age, risk score, and staging was constructed to assign scores and predict 1-, 3-, and 5-year survival probabilities (**Figure 5E**). Calibration curves indicated a high concordance between predicted and observed survival probabilities, reflecting satisfactory model performance (**Figure 5F**). ROC curve analysis further confirmed the model’s predictive accuracy, with AUC values of 0.747, 0.716, and 0.696 for 1-, 3-, and 5-year survival, respectively (**Figure 5G**). Based on the above analysis, this prognostic model demonstrates significant potential for assessing patient survival risk and guiding clinical decision-making. It provides physicians with individualized survival estimates and aids in the development of more targeted treatment strategies, ultimately enhancing the overall management of patient prognosis.

### Association analysis of risk scores with immune infiltration characteristics, molecular typing, TMB, and drug sensitivity

**Figure 6:**
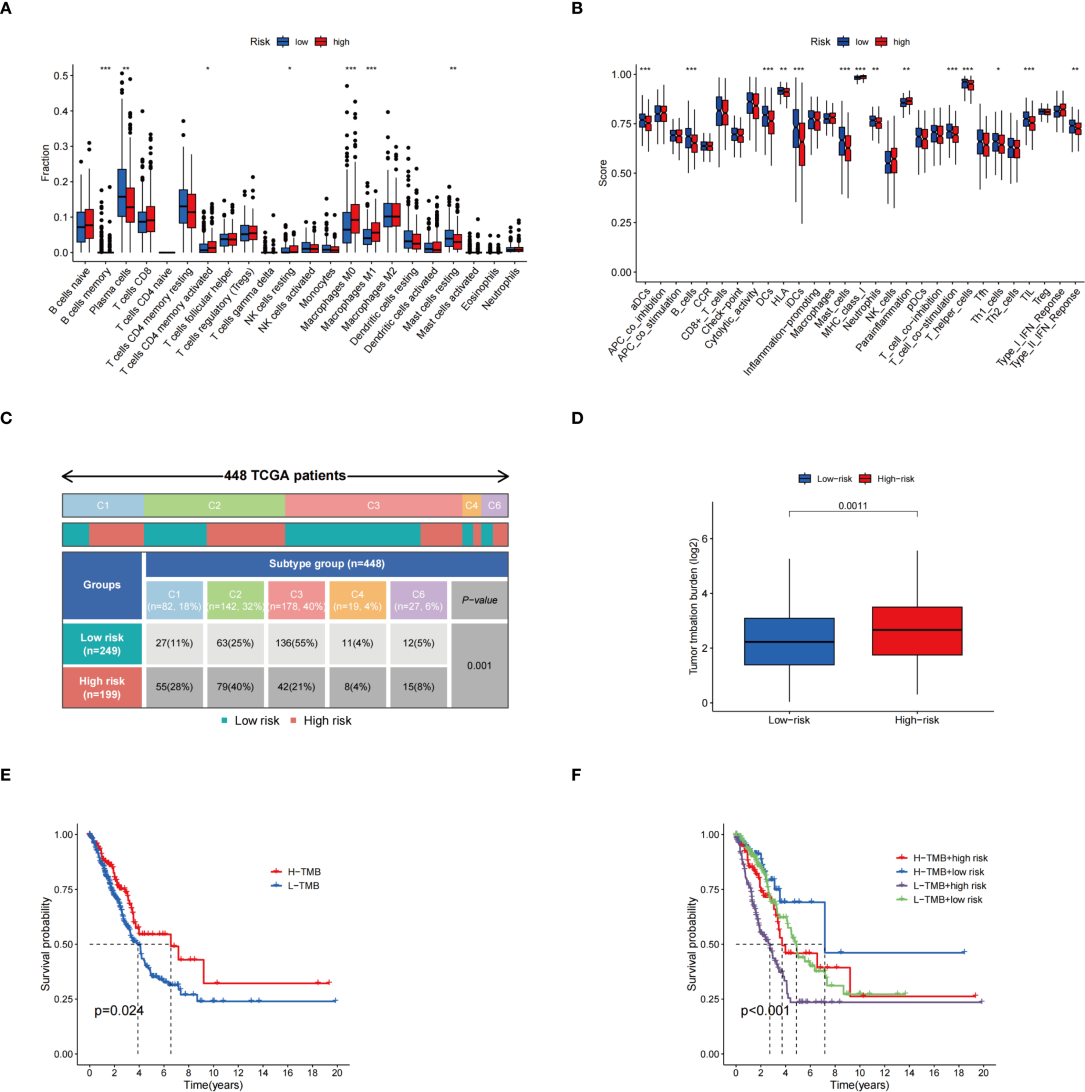
Correlation analysis of risk scores with immune infiltration characteristics, molecular typing, and TMB. **(A)** Demonstrating the differences in the proportions of different immune cell infiltrates in the two risk groups; **(B)** Comparison of immune-related functional scores assessed based on the ssGSEA method among different risk groups; **(C)** Distribution of TCGA patients among immune subtypes and the differences in their proportions in the two risk groups; **(D)** TME expression in the high-risk and low-risk groups; **(E)** KM survival curves for groups with different TMB expression levels; **(F)** KM curves for survival analysis based on combined grouping of risk score and TMB. ^*^p < 0.05; ^**^p < 0.01; ^***^p < 0.001.

Immune cell differential analysis revealed that memory resting mast cells, B cells, and plasma cells, were significantly more abundant in the low-risk group, whereas resting NK cells, activated CD4 memory T cells, and M0- and M1-type macrophages were enriched in the high-risk group (**Figure 6A**). Functional immune analyses indicated enhanced activity in B cells, activated dendritic cells, dendritic cells, HLA molecules, mast cells, infiltrating dendritic cells, neutrophils, T-cell co-stimulation, helper T cells, Th1 cells, tumor-infiltrating lymphocytes, and type II interferon responses within the low-risk group. Conversely, increased expression of MHC class I molecules and parainflammatory markers was observed in the high-risk group (**Figure 6B**). Immunophenotyping analysis demonstrated a statistically significant difference in immunophenotype distribution between different risk groups (**Figure 6C**). TMB was markedly elevated in the high-risk group compared to the low-risk group, as shown in **Figure 6D**. Survival analysis revealed that patients with high TMB had significantly improved survival probability relative to those with low TMB (p = 0.024) (**Figure 6E**). Further stratification combining risk status and mutation burden demonstrated that patients with high TMB and low risk exhibited the highest survival rates, while those with low TMB and high risk showed the lowest survival rates (**Figure 6F**). These results suggest that TMB and risk stratification jointly influence patient survival outcomes.

**Figure 7:**
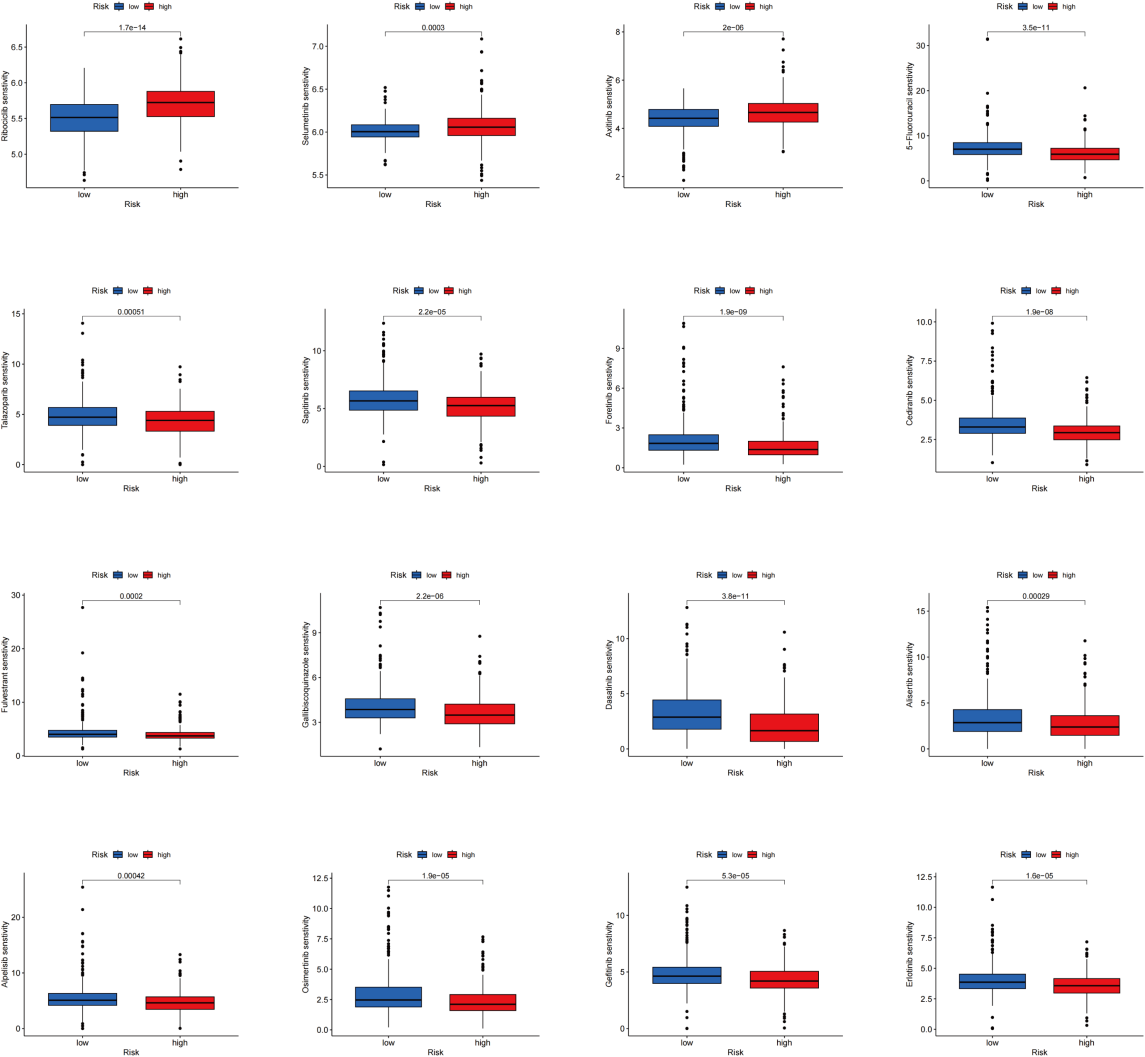
Results of drug sensitivity analysis between the two risk groups.

The differences in drug sensitivity between the two groups were further investigated. It was found that the low-risk group exhibited lower IC50 values for Ribociclib, Selumetinib, and Axitinib, indicating increased sensitivity to these drugs. Conversely, higher IC50 values were observed in the low-risk group for 5-Fluorouracil, Talazoparib, Sapitinib, Fenretinide, Cediranib, Fluvastatin, Galiellalactone, Dasatinib, Alisertib, Apitolisib, Osimertinib, Gefitinib, and Erlotinib, suggesting decreased sensitivity (**Figure 7**). These findings offer valuable guidance for selecting the most appropriate drugs based on patients’ risk scores in clinical practice, particularly in the context of precision medicine and targeted treatment strategies.

### ZDHHC5 expression and activity across various cancer types

**Figure 8:**
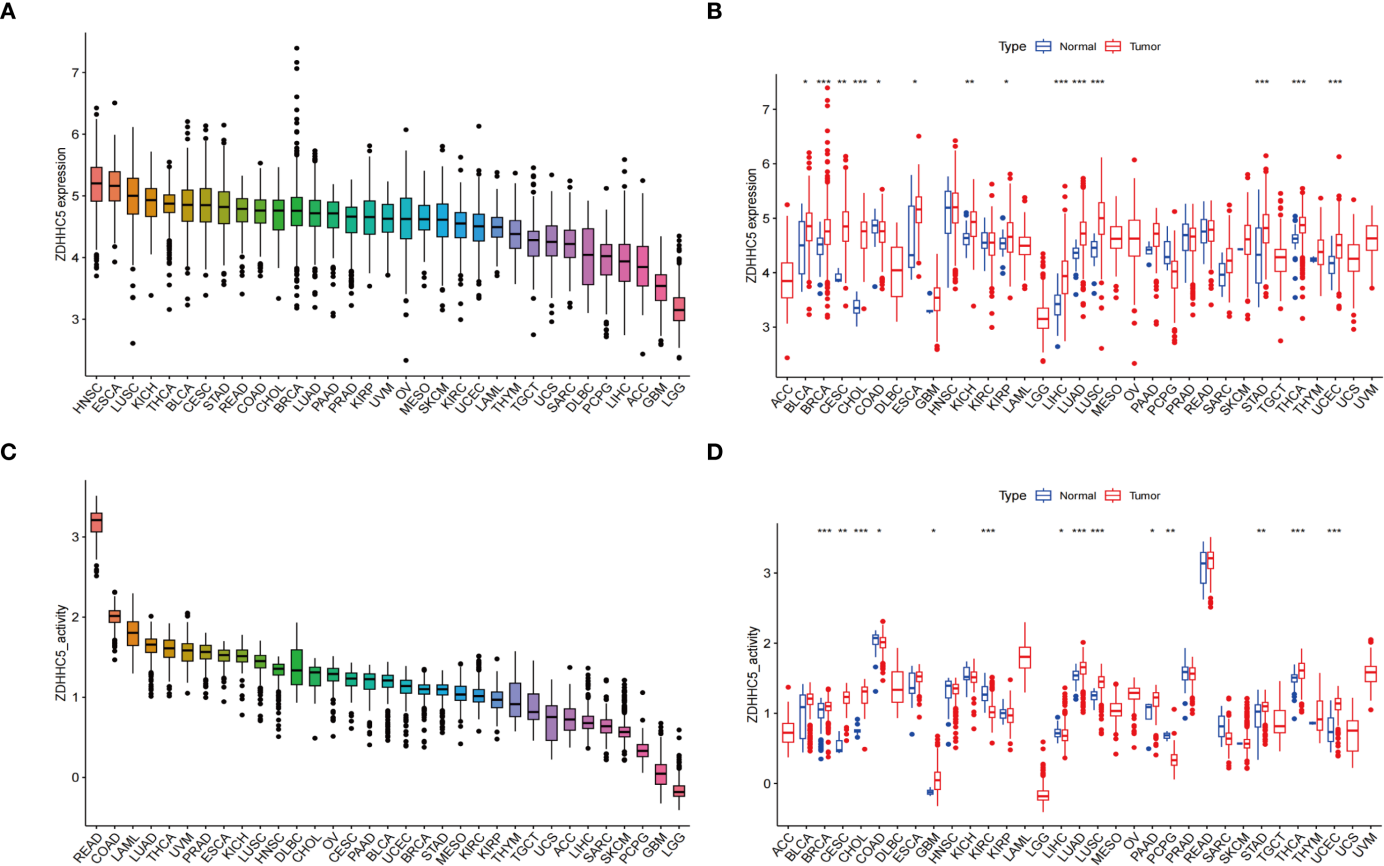
ZDHHC5 expression and activity across various cancer types. **(A)** ZDHHC5 expression in tumor tissues; **(B)** Expression of ZDHHC5 in tumor and normal samples; **(C)** ZDHHC5 activity in tumor tissues; **(D)** ZDHHC5 activity in tumor and normal samples. ^*^p < 0.05; ^**^p < 0.01; ^***^p < 0.001.

Next, the role of ZDHHC5, the most critical gene in the model, was further investigated in a pan-cancer context. ZDHHC5 expression across 33 tumor types was analyzed using the TCGA database, with tissues ranked from highest to lowest expression. The highest expression level was detected in head and neck squamous cell carcinoma (HNSC) (**Figure 8A**). Compared with normal tissues, significantly higher expression of ZDHHC5 was detected in 13 cancer types: invasive breast carcinoma (BRCA), bladder urothelial carcinoma (BLCA), cholangiocarcinoma (CHOL), cervical squamous cell carcinoma and endocervical adenocarcinoma (CESC), esophageal carcinoma (ESCA), kidney chromophobe (KICH), liver hepatocellular carcinoma (LIHC), LUAD, lung squamous cell carcinoma (LUSC), stomach adenocarcinoma (STAD), thyroid carcinoma (THCA), uterine corpus endometrial carcinoma (UCEC), and kidney renal papillary cell carcinoma (KIRP) (**Figure 8B**). In contrast, downregulated expression was observed in colon adenocarcinoma (COAD). The activity levels of ZDHHC5 across 33 tumor types are shown in **Figure 8C**, with the highest activity in rectal adenocarcinoma (READ) and the lowest in lower-grade glioma (LGG). **Figure 8D** presents the comparison of ZDHHC5 activity between tumor and normal tissues. Elevated activity was observed in BRCA, CESC, CHOL, glioblastoma multiforme (GBM), LUSC, LUAD, pancreatic adenocarcinoma (PAAD), STAD, UCEC, and THCA, while reduced activity was noted in LIHC, COAD, pheochromocytoma and paraganglioma (PCPG), and kidney renal clear cell carcinoma (KIRC). Additionally, IHC staining from the HPA database showed a significant increase in ZDHHC5 protein expression across eight tumor types compared to normal tissues (**Supplementary Figure S3**). These findings highlight the markedly elevated expression of ZDHHC5 in various cancers, suggesting its potential involvement in cancer progression.

### ZDHHC5 expression, survival, and enrichment analysis in pan-cancer analysis

**Figure 9:**
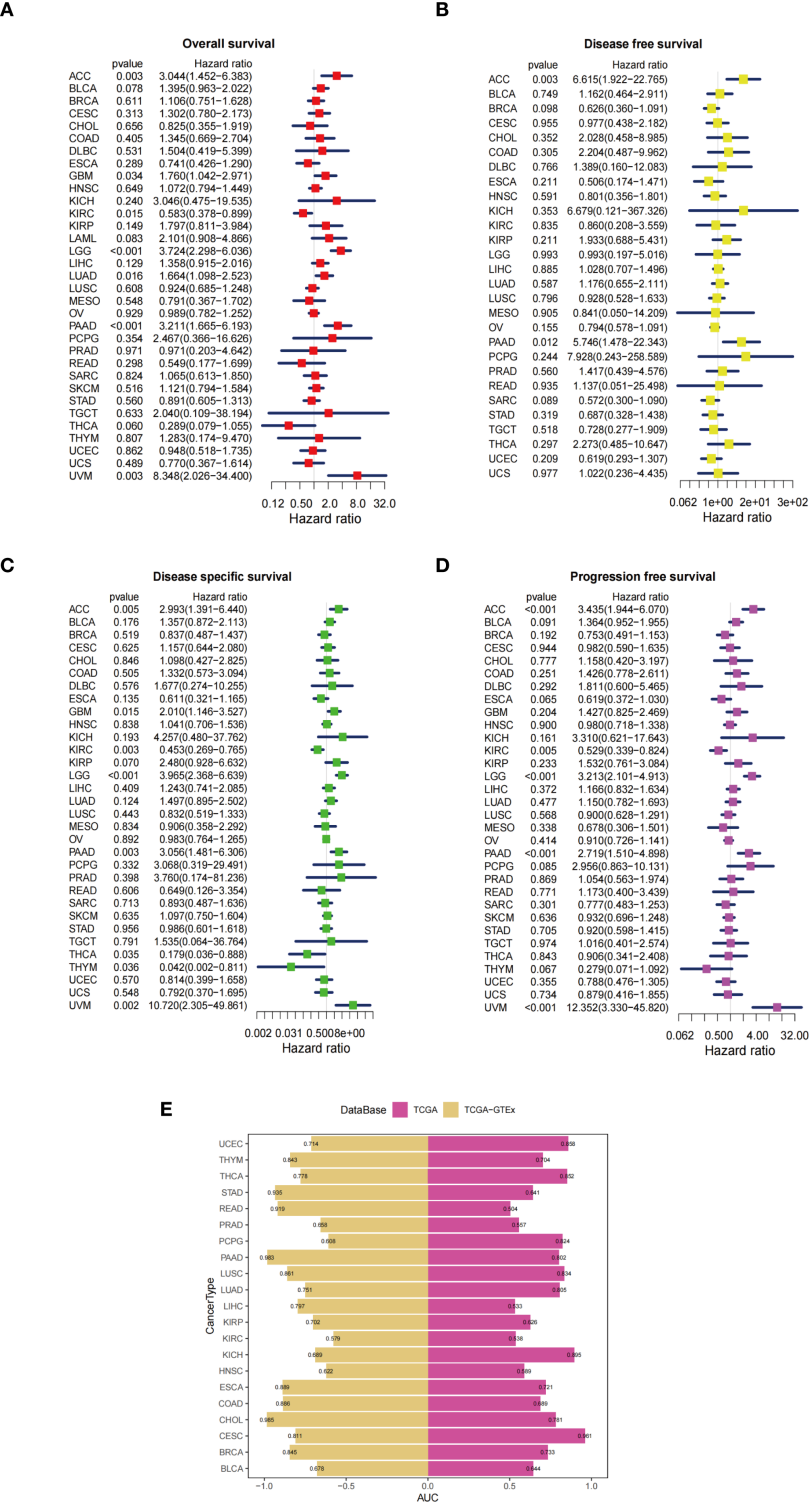
Forest plots and AUC values between ZDHHC5 and survival across multiple cancers. **(A)** The connection between ZDHHC5 and OS; **(B)** The connection between ZDHHC5 and DFS; **(C)** The connection between ZDHHC5 and DSS; **(D)** The connection between ZDHHC5 and PFS; **(E)** AUC values based on TCGA and GTEx databases across various types of cancer.

The association between ZDHHC5 expression and OS, disease-free survival (DFS), progression-free survival (PFS), and disease-specific survival (DSS) was investigated across various tumor types. Elevated ZDHHC5 expression was associated with reduced OS in adrenocortical carcinoma (ACC), PAAD, LUAD, uveal melanoma (UVM), LGG, and GBM (**Figure 9A**). In ACC and PAAD, high ZDHHC5 expression was also correlated with shorter DFS (**Figure 9B**). Furthermore, increased ZDHHC5 expression was associated with worse DSS outcomes in ACC, GBM, PAAD, UVM, and LGG (**Figure 9C**). High expression levels of ZDHHC5 were also inversely correlated with PFS in ACC, LGG, UVM, and PAAD (**Figure 9D**). Conversely, elevated ZDHHC5 expression in KIRC was positively associated with both OS and PFS (**Figures 9A, D**), and similarly, it correlated positively with DSS in KIRC, THCA, and thymoma (THYM) (**Figure 9C**). KM survival analysis further confirmed that patients with high ZDHHC5 expression had worse OS in PAAD, LUAD, LGG, UVM, and ACC (**Supplementary Figure S4A**), and poorer DFS in PAAD and ACC (**Supplementary Figure S4B**). Additionally, high ZDHHC5 expression was correlated with reduced DSS and PFS in PAAD, LGG, ACC, and UVM (**Supplementary Figures S4C, D**), while it was positively associated with OS and PFS in THCA and KIRC, respectively (**Supplementary Figures S4A, D**). The diagnostic utility of ZDHHC5 was also evaluated using TCGA and GTEx datasets (**Supplementary Figures S5**, **S6**). In TCGA, 12 tumor types had AUC values exceeding 0.7 (**Figure 9E**). In the GTEx dataset, 19 tumor types showed AUC values above 0.7, with PAAD, CHOL, STAD, and READ exhibiting AUC values greater than 0.9 (**Figure 9E**). These findings underscore the strong diagnostic potential of ZDHHC5 across a broad range of tumors. Moreover, the heterogeneous expression patterns and prognostic implications of ZDHHC5 in different cancers highlight its biological significance and potential clinical relevance as a biomarker for personalized cancer therapy.

GSEA and GO functional analyses demonstrated a significant association between ZDHHC5 and various immune-related functions in HNSC, COAD, uterine carcinosarcoma (UCS), LUSC, THCA, and STAD. However, enrichment patterns varied across tumor types. For example, STAD and THCA exhibited opposite enrichment trends, with negative enrichment observed in STAD and positive enrichment in THCA (**Supplementary Figure S7A**). These functions included detection of chemical stimuli, natural killer cell activation involved in immune responses, and regulation of defense responses to bacterial and fungal pathogens. GSEA, based on KEGG pathways, identified a positive correlation between ZDHHC5 and several immune-related signaling pathways in UCS, LIHC, UVM, LGG, LUSC, THYM, and GBM (**Supplementary Figure S7B**). These pathways included antigen processing and presentation, T-cell receptor signaling, toll-like receptor signaling, the intestinal immune network for IgA production, cytokine–cytokine receptor interactions, and natural killer cell-mediated cytotoxicity signaling pathway. These results further highlight the potential role of ZDHHC5 in regulating tumor immunity and provide a theoretical foundation for future research in cancer immunotherapy.

### ZDHHC5 and its association with immune infiltration, immune modulators, immune checkpoints, SNV-derived neoantigens, MSI, and TMB across cancers

**Figure 10:**
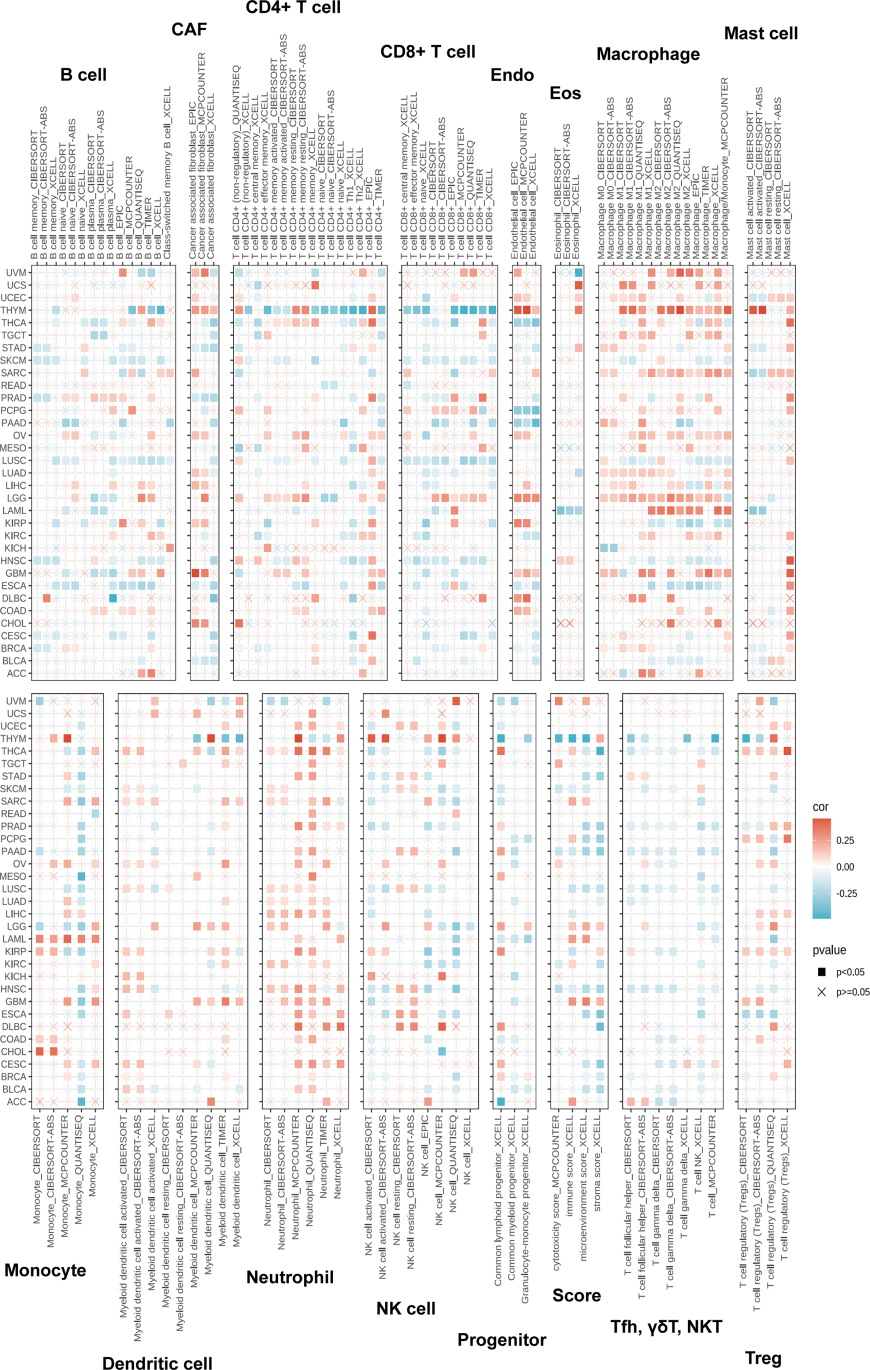
Relationship between various immune cell infiltrations and ZDHHC5 in different tumors.

Tumor immune cell infiltration is a key component of the tumor microenvironment (TME) and plays a critical role in determining tumor prognosis (25, 26). Accordingly, the association between ZDHHC5 expression and immune cell infiltration was investigated. **Figure 10** illustrates the positive correlation between ZDHHC5 expression and various immune-infiltrating cell types, including macrophages, neutrophils, cancer-associated fibroblasts, endothelial cells, mast cells, and monocytes. In most tumor types, ZDHHC5 expression was favorably associated with immune cell infiltration. However, in THYM, ZDHHC5 expression was negatively correlated with CD4^+^ and CD8^+^ T cells. In addition, **Figure 10** also presents the relationships between ZDHHC5 expression and immune, microenvironmental, and stromal scores across multiple cancer types. These findings further highlight the complex role of ZDHHC5 in modulating the tumor immune microenvironment and suggest potential mechanisms through which it may influence tumor progression.

The correlation between ZDHHC5 and common ICP genes was also investigated. In most tumor types, a strong connection was observed between ICP genes and ZDHHC5 expression (**Supplementary Figure S8A**). However, in THCA, skin cutaneous melanoma (SKCM), ESCA, CESC, HNSC, and LUSC, most ICP genes exhibited an inverse relationship with ZDHHC5 expression. According to data from the TISIDB database, ZDHHC5 expression across cancers was associated with immunostimulators, immunoinhibitors, and major histocompatibility complex (MHC) genes. Specifically, in GBM, mesothelioma (MESO), ovarian cancer (OV), UVM, testicular germ cell tumors (TGCT), sarcoma (SARC), UCS, and LGG, ZDHHC5 expression was positively correlated with immunostimulators, while negative correlations were observed in BLCA, ESCA, breast invasive carcinoma (BRCA), COAD, and LUSC (**Supplementary Figure S8B**). In addition, ZDHHC5 expression showed a positive association with immunoinhibitors in GBM, LGG, OV, TGCT, UCS, and SARC, whereas negative associations were observed in COAD, BRCA, LUSC, and PAAD (**Supplementary Figure S8C**). ZDHHC5 was also associated with MHC gene expression in ACC, CHOL, GBM, LGG, KIRC, OV, SARC, TGCT, THCA, UCS, and UVM. Conversely, negative correlations were identified in BRCA, BLCA, COAD, CESC, ESCA, LIHC, READ, LUSC, HNSC, LUAD, KIRP, and prostate adenocarcinoma (PRAD) (**Supplementary Figure S8D**). These findings further underscore the complexity and tumor-specific variability of ZDHHC5 in immune regulation.

SNVs are among the most common and widespread changes in the genome (27). **Supplementary Figure S8E** illustrates the levels of SNVs neoantigens in pan-cancer cells. TMB, which represents the total somatic mutation load in tumor cells (28), is a promising marker for assessing the response to immunotherapy (29). Our study demonstrated a favorable correlation between ZDHHC5 and TMB in UCEC, ACC, THYM, SARC, STAD, LUAD, LGG, and PAAD (**Supplementary Figure S8F**). Previous studies have established that MSI correlates with tumor prognosis (30). As shown in **Supplementary Figure S8G**, ZDHHC5 was strongly associated with MSI in UVM, KICH, KIRC, and UCEC, and inversely associated with MSI in ACC, BRCA, THCA, SKCM, HNSC, and diffuse large B-cell lymphoma (DLBC). These findings form the foundation for further investigation of its mechanism of action and potential clinical applications.

### ZDHHC5 expression, clinical characterization, and enrichment analysis in LUAD

**Figure 11:**
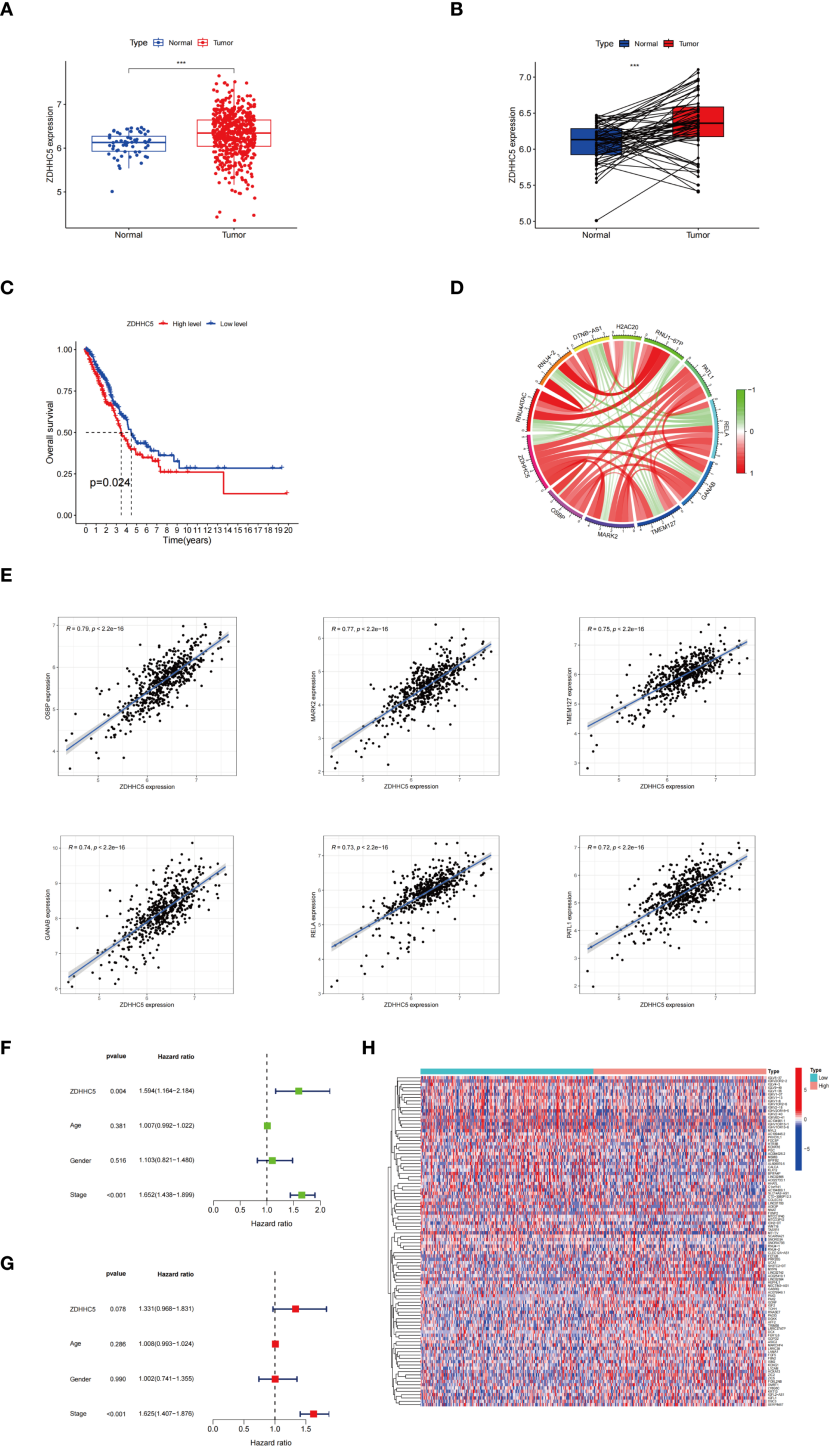
ZDHHC5 expression and clinical characterization in LUAD. **(A)** ZDHHC5 expression levels in LUAD versus normal samples; **(B)** ZDHHC5 expression levels in paired LUAD versus normal samples; **(C)** Connection between ZDHHC5 and OS; **(D)** Co-expression circle plot depicting the relationship between ZDHHC5 and 11 genes; **(E)** The six genes with the strongest positive correlation with ZDHHC5; **(F)** Univariate Cox regression analysis; **(G)** Multivariate Cox regression analysis; **(H)** The top 50 DEGs in the high and low ZDHHC5 expression groups. ^***^p < 0.001.

Given the significantly elevated expression of ZDHHC5 in LUAD and its strong associations with patient prognosis, the tumor immune microenvironment, and various ICP genes, a detailed investigation into its specific role in LUAD was conducted, considering the high incidence and clinical relevance of this cancer. ZDHHC5 expression was observed to be markedly elevated in LUAD samples (n = 541) compared to normal lung tissues (n = 59) (**Figure 11A**). Paired sample analysis further confirmed these results (**Figure 11B**). KM survival analysis indicated that patients with low ZDHHC5 expression had significantly longer OS than those with high expression (p = 0.024) (**Figure 11C**). Co-expression analysis identified genes strongly correlated with ZDHHC5 (|correlation coefficient| > 0.7, p < 0.001). The six most positively correlated genes were OSBP, MARK2, TMEM127, GANAB, RELA, and PATL1, all with correlation coefficients greater than 0.7 (**Figure 11D**). The five most negatively correlated genes included RNU4ATAC, RNU4-2, DTNB-AS1, H2AC20, and RNU1-67P (**Figure 11E**). Cox regression analysis validated that ZDHHC5 expression and clinical stage were independent prognostic factors in LUAD (**Figures 11F, G**). A total of 364 DEGs were detected between the high and low ZDHHC5 expression groups (**Supplementary Table S2**). Of these, 306 genes were upregulated in the high-expression group, while 58 genes showed increased expression in the low-expression group. Heatmaps of the top 50 DEGs in each group were generated to visualize these differences (**Figure 11H**). These results not only enhance our understanding of the role of ZDHHC5 in LUAD but also offer valuable insights for the development of future therapeutic strategies.

GO, KEGG, and GSEA enrichment analyses were performed to explore the potential regulatory mechanisms of ZDHHC5 DEGs. GO and KEGG analyses revealed significant associations of DEGs with 206 GO terms and 13 KEGG pathways (**Supplementary Tables S3** **, S4**). GO analysis alone revealed that the DEGs were predominantly involved in various biological processes, including filament organization, keratinization, and keratinocyte differentiation intermediates. Cellular components were enriched in desmosomes, connexin complexes, and cornified envelopes. For molecular functions, DEGs were enriched in structural constituents of the skin epidermis, voltage-gated monoatomic ion channel activity, and voltage-gated monoatomic cation channel activity (**Supplementary Figure S9A**). The most notable KEGG pathways included neuroactive ligand-receptor interactions, human papillomavirus infection, and the PI3K-Akt signaling pathway (**Supplementary Figure S9B**). GSEA demonstrated significant enrichment of cell cycle checkpoint functions in the ZDHHC5 high-expression group, suggesting a role for ZDHHC5 in regulating cell cycle pathways (**Supplementary Figures S9C, D**). Thus, a more comprehensive understanding of the molecular mechanisms of ZDHHC5 in LUAD can be obtained, providing a theoretical foundation for the development of future therapeutic strategies.

### Correlations between ZDHHC5, immune infiltration, and drug sensitivity in LUAD

**Figure 12:**
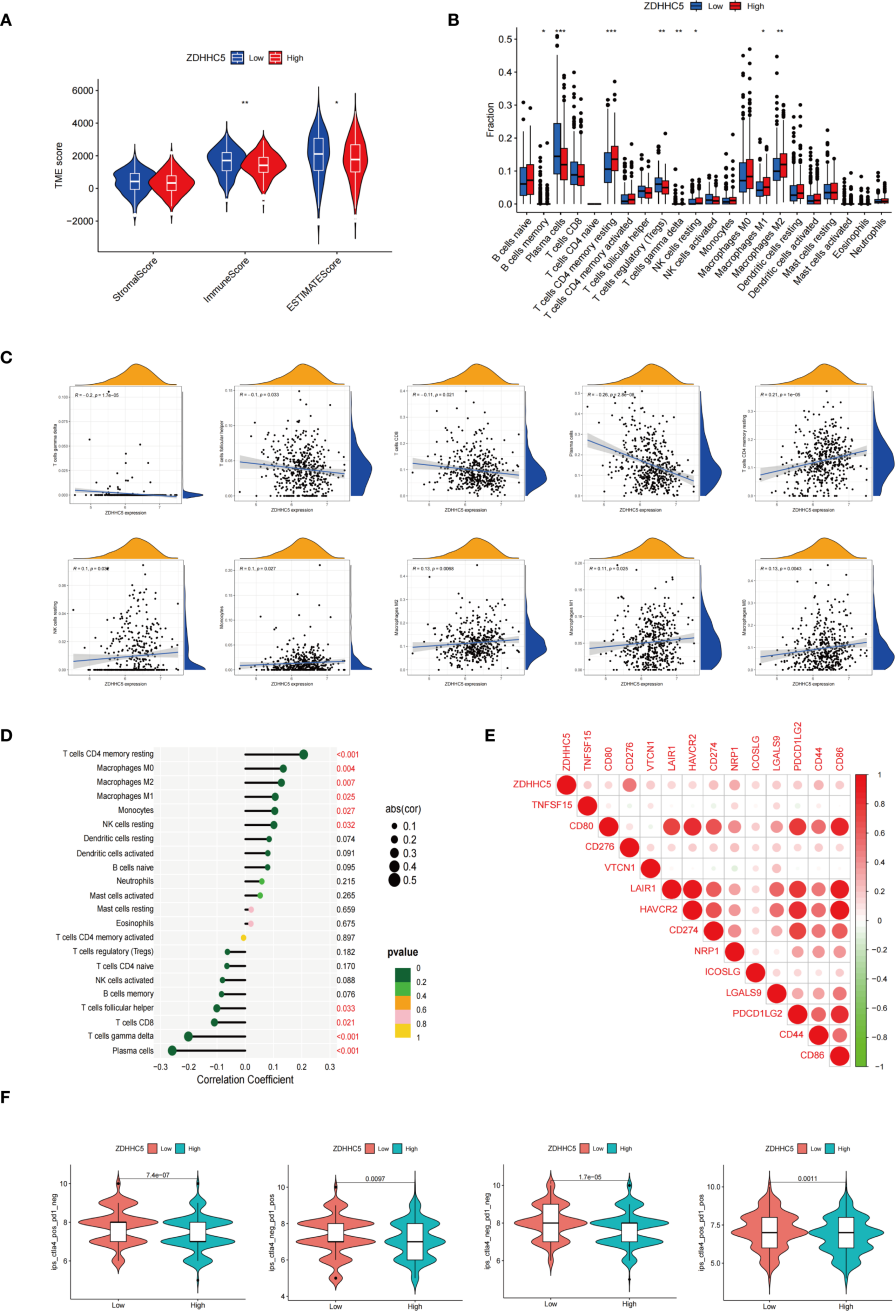
Correlations between ZDHHC5 and immune infiltration in LUAD. **(A)** Connection between ZDHHC5 and TME; **(B)** Effect of high and low ZDHHC5 expression on immune cell infiltration; **(C)** Connection graph between ZDHHC5 and immune cells; **(D)** Lollipop plot between ZDHHC5 and immune cells; **(E)** Correlation between ZDHHC5 and ICP genes; **(F)** Correlation between ZDHHC5 and immunotherapy. ^*^p < 0.05; ^**^p < 0.01; ^***^p < 0.001.

Further analyses of the TME were conducted. The ZDHHC5 low-expression group exhibited notably higher estimated and immune scores than the ZDHHC5 high-expression group (**Figure 12A**). Furthermore, we observed substantial disparities in the proportions of immune cells between the two groups, with eight out of 22 immune cell types showing notable differences (**Figure 12B**). Further correlation analyses revealed an association between ZDHHC5 expression and various immune cell types. ZDHHC5 showed a positive association with macrophages M0 (r = 0.13, p = 0.004), T cells CD4 memory resting (r = 0.21, p < 0.001), monocytes (r = 0.1, p = 0.027), macrophages M2 (r = 0.13, p = 0.007), macrophages M1 (r = 0.11, p = 0.025), and NK cells resting (r = 0.1, p = 0.032), while showing negative correlations with T cells follicular helper (r = -0.1, p = 0.033), T cells CD8 (r = -0.11, p = 0.021), T cells gamma delta (r = -0.2, p < 0.001), and plasma cells (r = -0.26, p < 0.001) (**Figures 12C, D**). Furthermore, we identified 13 ICP genes associated with ZDHHC5 (p < 0.001); CD276 had the highest correlation coefficient (COR = 0.49). Importantly, all ICP genes positively correlated with ZDHHC5 expression (**Figure 12E**). Finally, our analysis of ZDHHC5 expression and its association with immunotherapy demonstrated that patients with low ZDHHC5 expression showed improved efficacy across treatments with programmed death 1 (PD1) inhibitors alone, cytotoxic T-lymphocyte-associated protein 4 (CTLA4) inhibitors, or a combination of PD1 and CTLA4 inhibitors (**Figure 12F**). These observations indicate the significant role of ZDHHC5 in modulating immune cell infiltration, affecting responses to immunotherapy, and suggest potential clinical therapeutic avenues.

LUAD samples were classified into high and low expression groups based on ZDHHC5 levels to examine its correlation with drug sensitivity (**Supplementary Figure S10**). Analysis revealed that patients with low ZDHHC5 expression exhibited significantly reduced IC50 values for various drugs, including vorinostat, venetoclax, sabutoclax, ribociclib, PRIMA-1MET, obatoclax mesylate, niraparib, nilotinib, mitoxantrone, linsitinib, entinostat, doramapimod, dabrafenib, afuresertib, and leflunomide, suggesting enhanced sensitivity. Conversely, the high ZDHHC5 expression group demonstrated markedly lower IC50 values for cediranib, indicating increased drug sensitivity. These findings indicate that ZDHHC5 may have a pivotal role in modulating drug resistance, offering insights for developing personalized treatment approaches. Future research should delve into the molecular mechanisms by which ZDHHC5 influences drug metabolism and response, aiming to enhance the treatment outcomes and prognostic precision for LUAD.

### ZDHHC5 may promote the proliferation and metastasis of LUAD cells via the PI3K/AKT pathway

**Figure 13:**
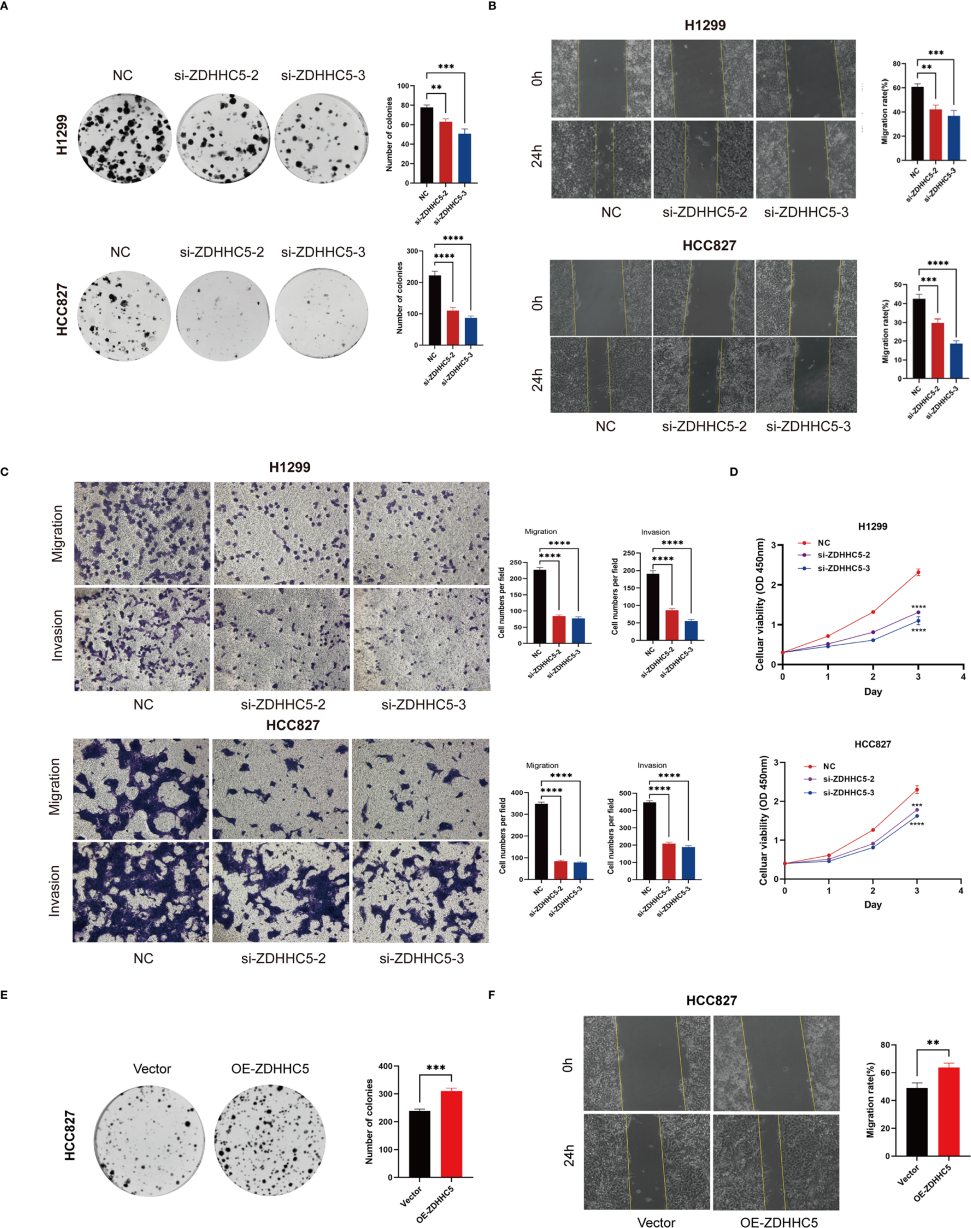
Impact of ZDHHC5 knockdown and overexpression in H1299 and HCC827 cells. **(A)** Colony formation assay to verify proliferation ability; **(B)** Wound healing assay to verify migration ability; **(C)** Transwell assay to verify migration and invasion ability; **(D)** CCK-8 assay to verify proliferation ability; **(E)** *Colony formation assay shows more colonies in OE-ZDHHC5 group;* **(F)** *Wound healing assay indicates higher migration rate in OE-ZDHHC5 group at 24 hours.* ^**^p < 0.01; ^***^p < 0.001; ^****^p < 0.0001.

Further experiments validated the overall function of ZDHHC5 in LUAD patients. According to data from the Cancer Cell Line Encyclopedia database, ZDHHC5 expression was relatively high in the H1299 and HCC827 cell lines (**Supplementary Figure S11**). As a key palmitoylation-related protein, ZDHHC5 has been studied in various cancer types, indicating its functional significance. It is hypothesized that the role of ZDHHC5 is not limited to LUAD. Therefore, both the LUAD cell line HCC827 and the NSCLC cell line H1299 were used for experimental validation. Our findings revealed that ZDHHC5 expression was significantly elevated in H1299 and HCC827 cells compared to normal lung epithelial BEAS-2B cells (**Supplementary Figure S12A**). The efficiency of ZDHHC5 knockdown was assessed via qRT-PCR and Western blotting, which demonstrated that both siRNA-2 and siRNA-3 effectively reduced ZDHHC5 mRNA and protein levels in both cell lines (**Supplementary Figures S12B, C**). Colony formation and CCK-8 assays showed that ZDHHC5 knockdown significantly inhibited the proliferative capacity of H1299 and HCC827 cells (**Figures 13A, D**). In addition, the invasion and migration abilities of cells following ZDHHC5 knockdown were evaluated using Transwell and wound healing assays. The results revealed that the migration and invasion abilities of tumor cells were significantly reduced after ZDHHC5 knockdown (**Figures 13B, C**).

Additionally, HCC827 cells were transfected with an overexpression plasmid (OE-ZDHHC5) and a control plasmid (vector) to overexpress ZDHHC5 (due to its low baseline expression in this cell line). Transfection efficiency was validated by qRT-PCR (**Supplementary Figure S12D**) and Western blotting (**Supplementary Figure S12E**). Colony formation assays showed that the number of colonies in the OE-ZDHHC5 group was significantly higher than in the vector group (**Figure 13E**). Moreover, wound healing assays indicated that the migration ability of OE-ZDHHC5 cells was significantly enhanced compared to the control group 24 hours after scratching (**Figure 13F**). These results suggest that overexpression of ZDHHC5 significantly enhances the proliferation and migration abilities of the HCC827 cell line.

**Figure 14:**
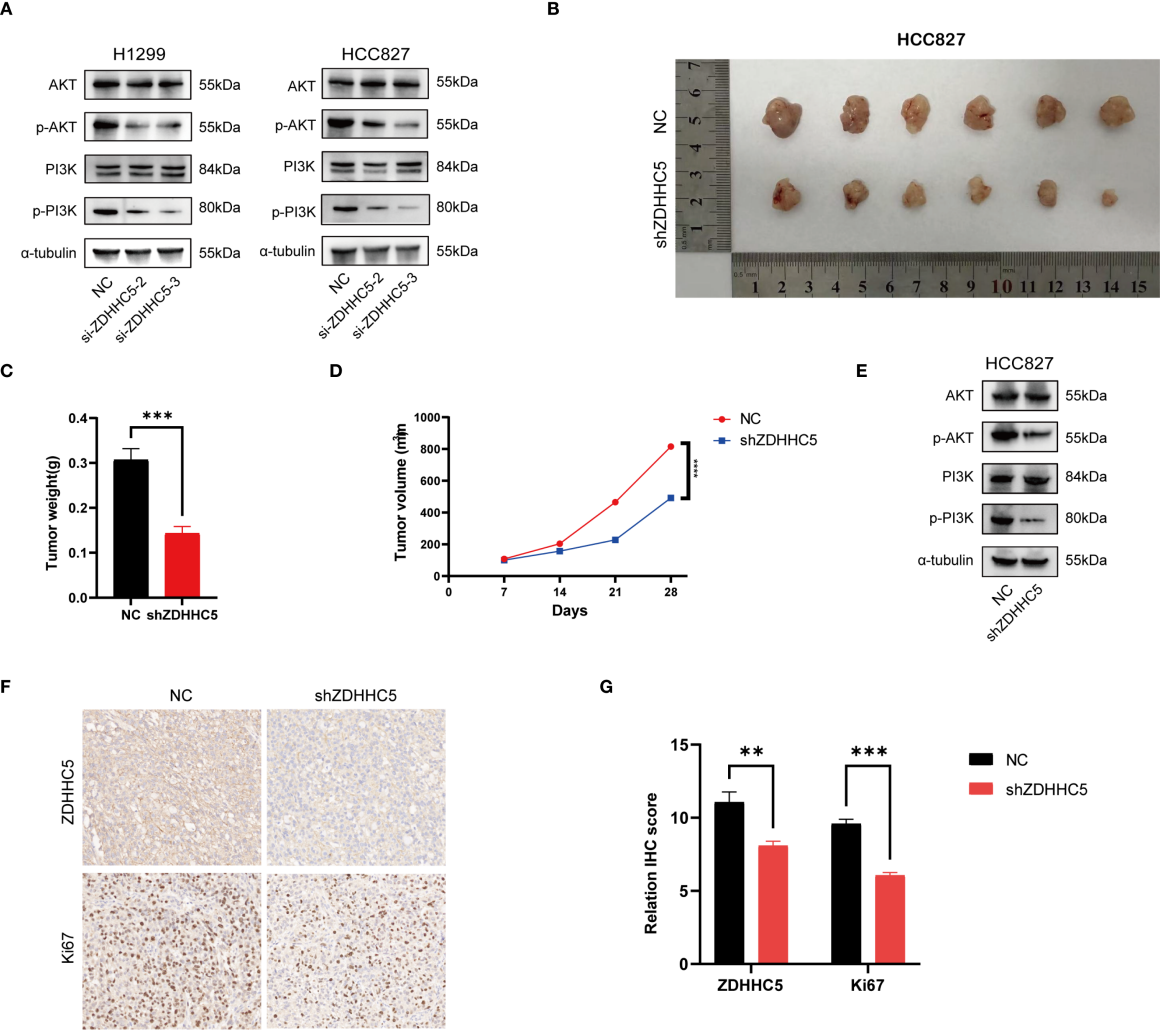
The effect of ZDHHC5 knockdown on tumor growth and the PI3K/AKT signaling pathway. **(A)** ZDHHC5 knockdown reduces the phosphorylation levels of AKT and PI3K; **(B)** HCC827 mouse xenograft tumor image; **(C)** Comparison of tumor weights between NC and shZDHHC5 groups; **(D)** Tumor growth curve; **(E)** Western blot results from HCC827 mouse model showing that shZDHHC5 inhibits the AKT/PI3K signaling pathway; **(F)** Immunohistochemical staining of ZDHHC5 and Ki67. Scale bars, 100 µm; **(G)** Comparison of ZDHHC5 and Ki67 immunohistochemical scores. ^**^p < 0.01; ^***^p < 0.001.

Disruption of the PI3K/AKT signaling pathway plays a critical role in regulating cell growth (31, 32). KEGG analysis demonstrated significant enrichment of ZDHHC5 within the PI3K/AKT pathway. To assess the impact of ZDHHC5, PI3K/AKT and its phosphorylated forms (p-AKT/p-PI3K) were analyzed by Western blotting following ZDHHC5 knockdown. The results indicated that ZDHHC5 knockdown substantially reduced the phosphorylation levels of AKT and PI3K (**Figure 14A**). To further investigate the relationship between ZDHHC5 and lung adenocarcinoma (LUAD) development *in vivo*, HCC827 cells were transfected with shZDHHC5 lentivirus, with shZDHHC5–3 showing the highest knockdown efficiency. These cells were then used for subsequent experiments (**Supplementary Figures S12F, G**). Transfected cells were injected into nude mice to establish xenograft tumor models. The results revealed that ZDHHC5 knockdown significantly reduced tumor volume and weight in the mice (**Figures 14B–D**). Western blot analysis further confirmed that ZDHHC5 knockdown markedly decreased the expression levels of p-AKT and p-PI3K in the tumor tissues (**Figure 14E**). Additionally, the expression of the cell proliferation marker Ki67 was significantly downregulated following ZDHHC5 knockdown (**Figures 14F, G**). In summary, ZDHHC5 knockdown may inhibit LUAD cell proliferation and metastasis by suppressing the PI3K/AKT signaling pathway.

## Discussion

LUAD, a common subtype of NSCLC, is characterized by high invasiveness and poor prognosis (33–35). As the incidence of LUAD continues to rise, it has become a significant public health challenge (36). Consequently, it is crucial to identify new therapeutic targets and prognostic biomarkers. Recent advances in molecular biology have highlighted the critical role of palmitoylation in cancer progression (18, 37). This study, therefore, aims to investigate the role of palmitoylation-related genes in LUAD and propose a prognostic prediction model based on these genes, with the goal of providing scientific guidance for clinical decision-making.

This study thoroughly examined the genomic variations, expression profiles, and their impact on patient prognosis of palmitoylation-related genes in LUAD, elucidating the role of these genes in LUAD. A multi-dimensional research strategy was employed, beginning with the identification of distinct gene expression subpopulations through consensus clustering analysis. This was followed by the construction of a prognostic risk model based on SHAP analysis to assess the contribution of each gene. Additionally, the connections between risk scores, immune infiltration characteristics, TMB, and drug sensitivity were explored. In particular, the key gene ZDHHC5 was the focus of the model. Its expression in various cancers, along with its associations with prognosis, immune cell infiltration, ICP genes, immune regulatory factors, MSI, TMB, and drug sensitivity were analyzed. Functional enrichment analysis delved deeper into the link between ZDHHC5, LUAD prognosis, and immune response. Experimental validation suggests that ZDHHC5 may promote LUAD cell proliferation, invasion, and migration through the PI3K/AKT pathway. In conclusion, through experimental validation and multi-omics analysis, the multifaceted roles of ZDHHC5 in cancer were revealed. These findings not only enhance the accuracy of diagnostic markers but also provide a theoretical basis for targeted therapy.

The palmitoleic acid-modified gene prognostic model developed in this study demonstrated significant advantages in immune microenvironment and prognostic stratification. The model accurately distinguished between low-risk and high-risk groups, with the low-risk group enriched in memory quiescent mast cells, B cells, and plasma cells, accompanied by enhanced B cell activation and type II interferon response. In contrast, the high-risk group was enriched in quiescent NK cells and M0/M1 macrophages, and exhibited high expression of MHC-I molecules. This analysis overcomes the limitations of traditional mutation/metabolic models, which only quantify cell abundance, and provides a more in-depth understanding of immune microenvironment characteristics. Additionally, risk scores significantly differentiated survival rates across all subgroups stratified by gender, age, and stage. Univariate and multivariate Cox regression analyses indicated that the HR for risk scores was the highest, outperforming the traditional TNM staging system, with patients in the high-risk group and high TMB exhibiting the poorest survival outcomes. By integrating risk scores with clinical variables, a nomogram was constructed, improving the AUC values for 1-, 3-, and 5-year OS to 0.747, 0.716, and 0.696, respectively, thereby providing a direct tool for personalized treatment. This model addresses the limitations of existing tools by linking palmitoleic acid gene expression with the functional state of the immune microenvironment, resolving the inability to dynamically elucidate immune suppression mechanisms and the differing prognoses among patients at the same stage. These findings further highlight its significant potential for clinical translation.

The risk model based on a five-gene signature has shown considerable potential for clinical applications. The predictive model developed integrates gene expression data and survival information, effectively predicting patient prognosis and providing a scientific foundation for the formulation of personalized treatment strategies. In clinical practice, qRT-PCR and NGS detection panels are essential tools for gene detection. qRT-PCR technology is sensitive, simple, cost-effective, and suitable for routine clinical testing, providing rapid results (38). In contrast, NGS technology offers higher throughput and greater accuracy (39). Although NGS incurs higher equipment and operational costs, it delivers more comprehensive information, particularly in precision medicine and personalized treatment, by identifying genes associated with tumor progression. From a cost-effectiveness standpoint, while qRT-PCR has advantages in cost, NGS holds greater potential when high-throughput, multi-gene simultaneous detection is required. As technology progresses and equipment costs decline, the feasibility and accessibility of NGS in clinical settings will continue to improve. By combining the strengths of qRT-PCR and NGS, particularly in cancer screening and risk prediction, more personalized and precise diagnostic and treatment strategies can be achieved.

The immune microenvironment plays an integral role in the progression of LUAD (40, 41). By analyzing the differences in immune infiltration between two palmitoylation-related groups, significant variations in immune infiltration characteristics were observed. The group B exhibited higher proportions of monocytes, CD4 memory resting T cells, and CD8 T cells, suggesting that patients in this group may have stronger antitumor immune responses, which could help explain their better prognosis. In contrast, group A was characterized by elevated levels of naïve B cells and activated CD4 memory T cells, potentially reflecting weaker immune responses that may promote tumor progression. Differential expression of HLA genes further revealed immune escape mechanisms (42, 43), with multiple HLA genes associated with enhanced antigen presentation being upregulated in group B, thereby promoting T cell-mediated cytotoxicity. The analysis of somatic mutations in this study revealed the genomic characteristics of LUAD and their association with the expression of palmitoylation-related genes. Notably, compared to the better-prognosed group B, group A exhibited higher mutation frequencies and was characterized by elevated TP53 mutation levels. This difference in mutation frequency suggests that mutational burden may play an essential role in tumor progression and patient prognosis (44). The higher mutation rate in group A may indicate that these tumors exhibit a more aggressive phenotype, which could lead to increased genomic instability and alterations in cellular pathways, thereby promoting tumorigenesis. Furthermore, the correlation between somatic mutations and immune cell infiltration highlights the complex interplay between the TME and host immune responses. group B exhibited higher levels of CD8 T cells and other immune cell infiltration, which are often associated with enhanced antitumor immunity. In contrast, group A was characterized by elevated levels of naïve B cells and activated CD4 T cells, which may reflect reduced immune response efficiency, further emphasizing the potential influence of somatic mutations on immune escape mechanisms.

A prognostic risk model constructed using genes associated with palmitoylation-related pathways revealed significant differences in sensitivity to multiple chemotherapy drugs between low-risk and high-risk groups, highlighting the potential for developing personalized treatment strategies based on individual risk profiles. Specifically, the low-risk group exhibited lower IC50 values for ribociclib, selumetinib, and axitinib, indicating greater sensitivity to these targeted therapies. This suggests that patients with a better prognosis may benefit from more aggressive treatment regimens incorporating these drugs. In contrast, the high-risk group demonstrated higher IC50 values for several traditional chemotherapy drugs (such as 5-fluorouracil, talazoparib, and osimertinib), suggesting potential resistance to these agents. The observed resistance in the high-risk group emphasizes the need to explore alternative treatment strategies, including combination therapy or novel drugs, to overcome existing resistance mechanisms. Overall, the correlation between risk scores and drug sensitivity underscores the importance of personalized medicine in the treatment of LUAD. By integrating genomic sequencing and risk stratification, clinicians can optimize treatment regimens, improve outcomes, and reduce unnecessary toxic side effects.

Differential expression of palmitoylation-related genes, particularly ZDHHC5, ZDHHC12, LYPLA1, and PPT2, underscores the potential roles of these genes in the pathogenesis and prognosis of LUAD. ZDHHC5, identified as the key gene in our risk model, will be discussed in detail later. ZDHHC12 has been shown to be involved in tumor progression. Studies have demonstrated that ZDHHC12-mediated claudin-3 palmitoylation plays a decisive role in OV progression (45). Research on LYPLA1 indicates that its inhibition can suppress the proliferation and migration of NSCLC cells (46). Although PPT2 has received limited attention in LUAD research, previous studies have shown its use in constructing an OV prognosis model based on genes related to mitochondrial metabolism (47). In summary, the expression patterns and functional significance of ZDHHC5, ZDHHC12, and PPT2 in LUAD not only enhance our understanding of the molecular mechanisms underlying this malignancy but also provide new avenues for developing targeted therapies to improve patient outcomes. Future studies should focus on elucidating the precise mechanisms by which these genes regulate tumor behavior and assess their potential as therapeutic targets for LUAD.

ZDHHC5, a member of the ZDHHC protein family (15), plays a crucial role in protein palmitoylation, directly affecting protein stability, localization, and interactions (45, 48). However, its role varies across cancer types. In gliomas and pancreatic cancer, ZDHHC5 promotes tumorigenesis and progression by regulating oncogenes and tumor suppressor genes (13, 49). This study found that ZDHHC5 overexpression enhances tumor cell proliferation and metastasis by activating PI3K/AKT and other pathways associated with tumor progression, driving malignant transformation. Additionally, ZDHHC5 is critical in cancer immune escape. As a key enzyme regulating PD-L1 stability, ZDHHC5 enhances PD-L1 stability via palmitoylation, promoting PD-L1-mediated immune suppression, which may exacerbate tumor growth and metastasis, leading to poor prognosis (50). In KIRC, ZDHHC5’s role differs significantly from other tumors. High ZDHHC5 expression is associated with better prognosis, showing significant positive effects on OS and PFS. ZDHHC5 may improve the tumor microenvironment and regulate fatty acid metabolism, leading to better prognosis. KIRC, a tumor with high immune infiltration (51), may benefit from ZDHHC5’s role in immune cell infiltration and function. As a regulator of fatty acid metabolism, ZDHHC5 may inhibit lipid accumulation in KIRC by modulating fatty acid uptake, distribution, oxidation, and storage, further improving prognosis. In summary, the opposite prognostic effects of ZDHHC5 in KIRC and LUAD are likely related to its distinct mechanisms in different tumor microenvironments, the signaling pathways it regulates, and the unique functions of its fatty acid palmitoylation targets. These findings provide important directions for future research.

Tumor-infiltrating immune cells significantly influence cancer treatment efficacy and patient prognosis (52, 53). Limited studies have investigated ZDHHC5 and immune infiltration in cancer; therefore, our research offers a novel perspective on the role of ZDHHC5 in the TME. Our research highlighted a meaningful relationship between ZDHHC5 and immune cell infiltration in various types of cancer. Specifically, ZDHHC5 expression positively correlated with macrophage and neutrophil infiltration, both of which are critical in the TME for promoting angiogenesis, inflammation, and tumor progression (54–56). Conversely, in THYM, ZDHHC5 expression negatively correlated with CD4+ and CD8+ T cells, which are crucial for anti-tumor immunity (57–59). The positive correlation between ZDHHC5 and macrophages and neutrophils suggests that ZDHHC5 may foster a tumor-promoting microenvironment by regulating cytokine production and immune cell recruitment. This hypothesis is consistent with our GSEA results, which indicate that ZDHHC5 participates in immune-related pathways, including cytokine-cytokine receptor interactions, and antigen processing and presentation. These findings imply that high ZDHHC5 expression facilitates immune evasion mechanisms, thereby driving tumor growth and progression. Additionally, the negative correlation between ZDHHC5 and CD4+ and CD8+ T cells in THYM suggests the potential immunosuppressive role of ZDHHC5 in certain cancers. CD4+ and CD8+ T cells are pivotal in orchestrating an effective antitumor response (60, 61), and their reduced infiltration into tumors with high ZDHHC5 expression may indicate impaired immune surveillance, potentially leading to resistance to T cell-mediated immunotherapy. Previous studies have shown that MSI and TMB have a substantial effect on the response and prognosis of patients with cancer undergoing immunotherapy (62, 63). Our results highlighted that ZDHHC5 expression exhibited a significant relationship with MSI and TMB in various tumors, and further ***in vivo*** experiments are required to confirm the impact of ZDHHC5 on TMB. ICP gene expression can influence the efficacy of immunotherapy in different cancers (64–67). Our study uncovered that most ICP genes were positively related to ZDHHC5 in most tumors. We further investigated the correlation between ZDHHC5 and immune modulators, including immunostimulators, immunoinhibitors, and MHCs, and found that ZDHHC5 strongly correlated with multiple immune modulators in different cancers. In conclusion, our results showed the varied role of ZDHHC5 in regulating the TME. Understanding the impact of ZDHHC5 on immunity may pave the way for new targeted therapies to modulate the immune environment in cancers.

The clinical translational potential of ZDHHC5 as a therapeutic target is increasingly recognized. Numerous studies have highlighted its critical role in tumors and metabolic diseases, particularly in the regulation of cancer cell proliferation, immune evasion, and various cellular physiological processes. Several compounds have been identified as potential inhibitors of ZDHHC5-mediated palmitoylation, demonstrating promising therapeutic prospects. For instance, docosahexaenoic acid (DHA) inhibits ZDHHC5 activity, promotes PD-L1 degradation, and exerts immune-enhancing effects (68). The LXR agonist T0901317 exhibits anti-proliferative effects in breast cancer cell models (69). Lomitapide has shown anti-tumor effects in pancreatic cancer animal models (13). Although 2-bromopalmitate (2-BP) has been used in preclinical studies as a broad-spectrum inhibitor to suppress tumorigenesis, its lack of specificity limits its clinical applicability (70–72). These findings provide a foundation for the development of selective ZDHHC5 small-molecule inhibitors. Innovative delivery strategies also present new opportunities for ZDHHC5-targeted therapy. Targeted delivery systems, such as antibody-drug conjugates or nanoparticles, can direct drugs precisely to tumor tissues while minimizing off-target effects on normal tissues. However, off-target effects remain a challenge in ZDHHC5-targeted therapy. Due to the high conservation of the DHHC motif among ZDHHC family members, achieving selectivity for a single isoenzyme through competitive inhibition is difficult, leading to off-target effects with broad-spectrum inhibitors like 2-BP (73). ZDHHC5 plays significant roles in the central nervous system, fatty acid metabolism, tumorigenesis, and cardiac function (50). Therefore, inhibiting ZDHHC5 may have adverse effects on cardiac and neural tissues. To mitigate off-target effects, future strategies may focus on targeting substrate recruitment sites, employing PROTAC degradation technology, utilizing targeted delivery systems, and exploring combination therapies. These approaches hold promise for improving treatment specificity and efficacy while minimizing side effects. Future research should prioritize the development of highly specific, low-toxicity inhibitors and assess their pharmacokinetics, pharmacodynamics, and safety to facilitate their clinical application.

Through *in vitro* and *in vivo* experiments, we found that ZDHHC5 may promote LUAD proliferation and metastasis via the PI3K/AKT pathway. The underlying mechanism may be as follows: First, the regulation of ZDHHC5 is closely associated with palmitoylation, which plays a crucial role in the activation, stability, and function of PI3K, AKT, and related molecules such as EGFR and mTOR. Specifically, PI3K, AKT, and other key molecules, including EGFR and mTOR, can be regulated by palmitoylation, thereby influencing the activity of the PI3K/AKT pathway and cellular biological behavior. Palmitoylation of AKT is particularly critical for its function and subcellular localization. AKT undergoes S-palmitoylation at the Cys344 residue, a modification that facilitates its translocation from the cytoplasm to the plasma membrane, a necessary step for its activation. Mutations at Cys344 lead to reduced phosphorylation at key sites (e.g., T308 and T450), impairing AKT function in processes such as autophagy (74). Thus, AKT palmitoylation plays a pivotal role in maintaining its functional stability and activity in cellular signaling. Although there is currently no direct evidence indicating that PI3K itself undergoes palmitoylation, existing literature suggests that palmitoylation may regulate AKT signaling through indirect mechanisms (75). For instance, ZDHHC22-mediated palmitoylation of mTOR reduces AKT signaling in breast cancer cells. Therefore, although further validation is needed to confirm whether PI3K is directly modified by palmitoylation, it is reasonable to speculate that PI3K may also be regulated by palmitoylation through indirect mechanisms. Moreover, palmitoylation of molecules such as EGFR and mTOR is critical for the regulation of the PI3K/AKT pathway. Palmitoylation of EGFR promotes the recruitment of PI3K, thereby activating the downstream AKT signaling pathway. Studies have shown that ZDHHC20 regulates the activation of the PI3K/AKT signal by palmitoylating EGFR and its subunits (76). When EGFR palmitoylation is inhibited, PI3K recruitment decreases, leading to a significant reduction in AKT phosphorylation and affecting cell proliferation. Palmitoylation of mTOR, mediated by ZDHHC22, reduces AKT signaling in breast cancer cells (77), further suggesting that mTOR palmitoylation may influence AKT activation. Additionally, palmitoylation of PCSK9 also plays a role in regulating the PI3K/AKT pathway. In liver cancer, palmitoylation of PCSK9 enhances its binding to PTEN, leading to PTEN degradation and relieving its inhibitory effect on AKT signaling (78). This mechanism suggests that ZDHHC5 may further impact AKT signaling by regulating the interaction between PCSK9 and PTEN. In summary, ZDHHC5 may precisely regulate the activation of the PI3K/AKT signaling pathway by palmitoylating key molecules such as AKT, EGFR, and mTOR, thereby influencing tumor cell proliferation and metastasis. Additionally, ZDHHC5’s regulation of the interaction between PCSK9 and PTEN may further impact AKT signaling. However, the specific mechanisms require further experimental validation.

Although this study provides a comprehensive analysis, several limitations should be noted. First, reliance on publicly available datasets introduces potential batch effects and variability due to differences in data collection and processing methods. Second, while the sample size is large, it may still be insufficient to fully capture the diversity of cancer types and patient responses. Third, the study lacks experimental validation of the underlying mechanisms. Finally, although the relationship between ZDHHC5 and various clinical and molecular characteristics was explored, its precise role in cancer progression and prognosis remains unclear. Future research will involve patient recruitment from multiple clinical centers, with samples collected from diverse geographical locations and population backgrounds, to further investigate the association between ZDHHC5 expression and treatment response. Additionally, our findings will be validated using humanized models, such as PDX mice and organoids, as well as additional LUAD cell lines. Mechanistic studies will incorporate CO-IP and mass spectrometry to identify the palmitoylation substrates of ZDHHC5 in LUAD cells, with particular attention to whether key molecules in the PI3K/AKT pathway undergo palmitoylation. We will also explore how ZDHHC5 regulates the PI3K/AKT pathway through this mechanism. These experiments aim to address the current limitations and further strengthen the generalizability of our findings.

## Conclusions

In brief, our findings underscore the essential role of palmitoylation-related genes, particularly ZDHHC5, in the pathogenesis and prognosis LUAD. The differential expression and genomic variations of these genes suggest their potential as biomarkers for patient risk stratification and treatment guidance. Consensus clustering analysis revealed significant immune-related differences between subgroups, indicating that alterations in the immune microenvironment may influence tumor behavior and patient prognosis. Furthermore, a robust prognostic model constructed using SHAP analysis emphasizes the importance of ZDHHC5 and its associated genes in predicting survival probability. Our correlation analysis further elucidated the complex relationships between risk scores, immune infiltration, TMB, and drug sensitivity, suggesting that ZDHHC5 may serve not only as a prognostic marker but also as a potential therapeutic target. In conclusion, these findings offer new insights into the biology of LUAD and may inform future clinical strategies.

## Data Availability

The original contributions presented in the study are included in the article/**Supplementary Material**. Further inquiries can be directed to the corresponding authors.
