# Supplementary figures and images for: ZDHHC5 as a central regulator in a palmitoylation-associated prognostic model for lung adenocarcinoma: insights from pan-cancer and experimental analyses
Source: Front Immunol. 2025 Sep 22;16:1643112. doi: 10.3389/fimmu.2025.1643112 (PMC12497774; doi:10.3389/fimmu.2025.1643112)

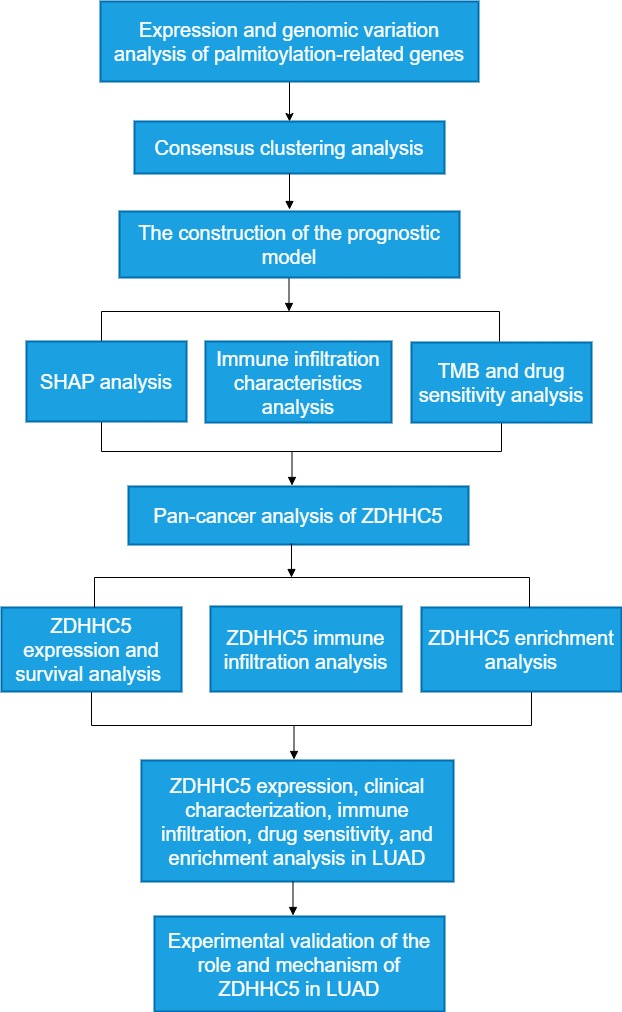

Supplement: Supplementary file 1 [file Image1.jpeg]

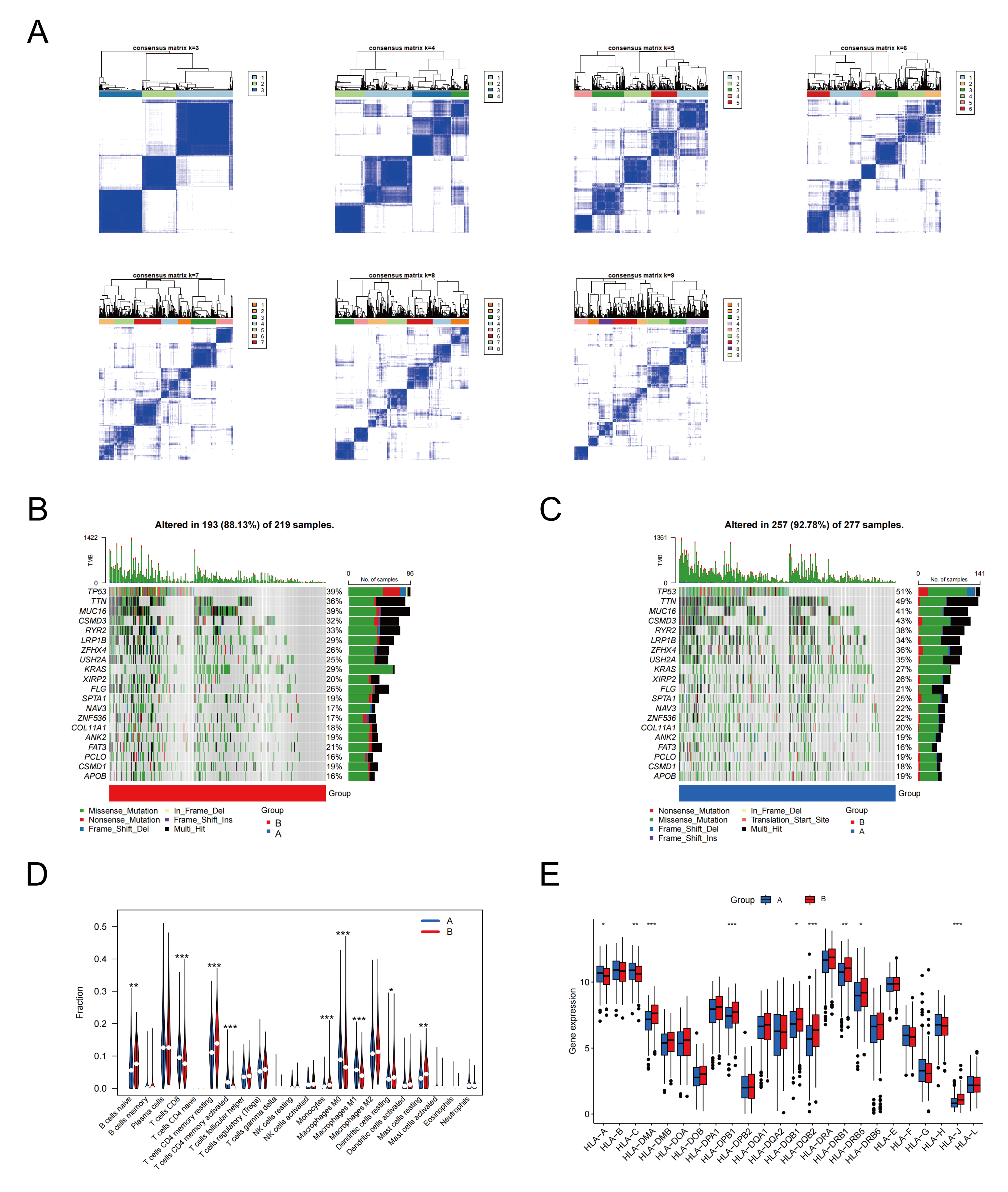

Supplement: Supplementary file 2 [file Image2.tif]

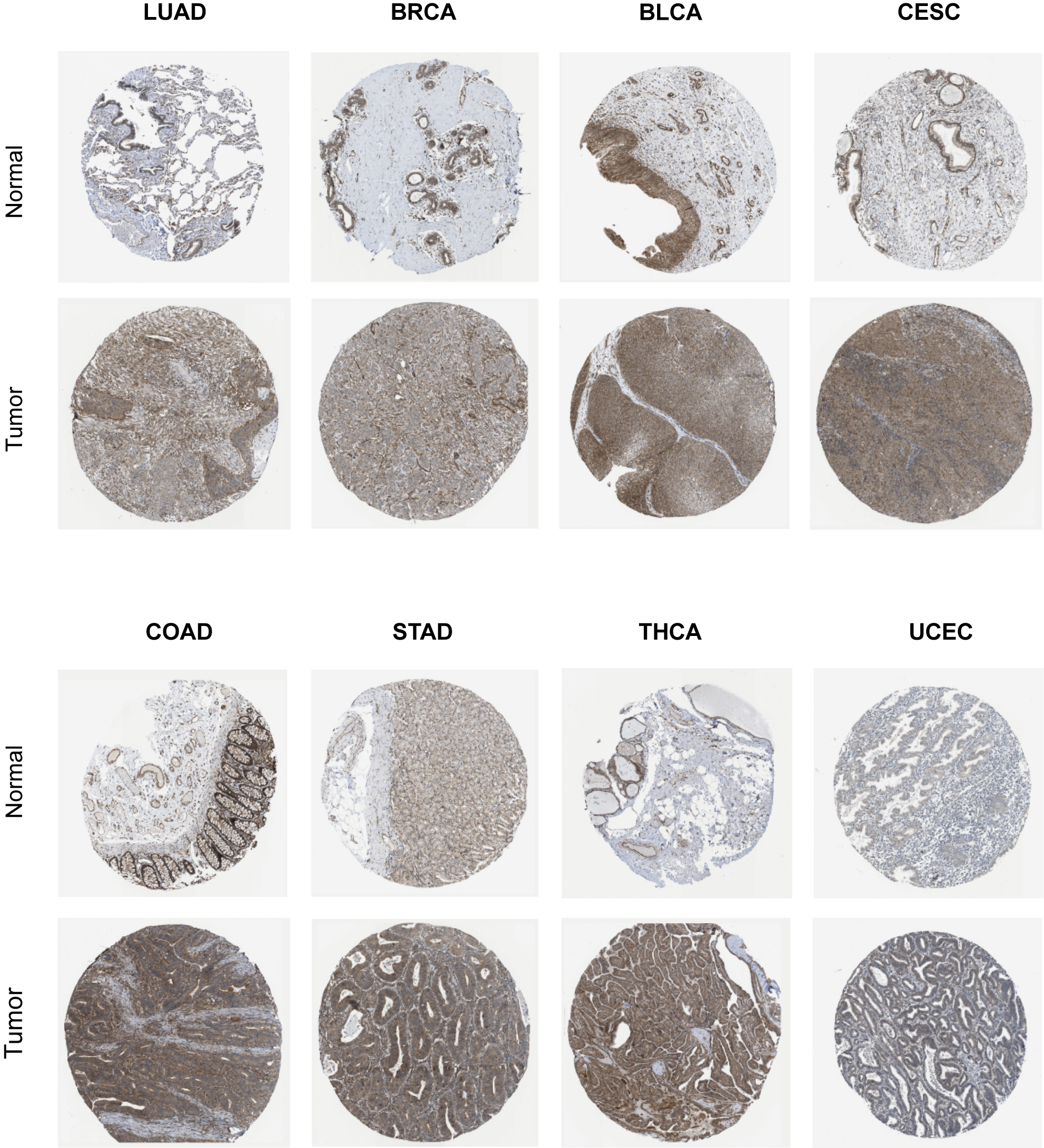

Supplement: Supplementary file 3 [file Image3.tif]

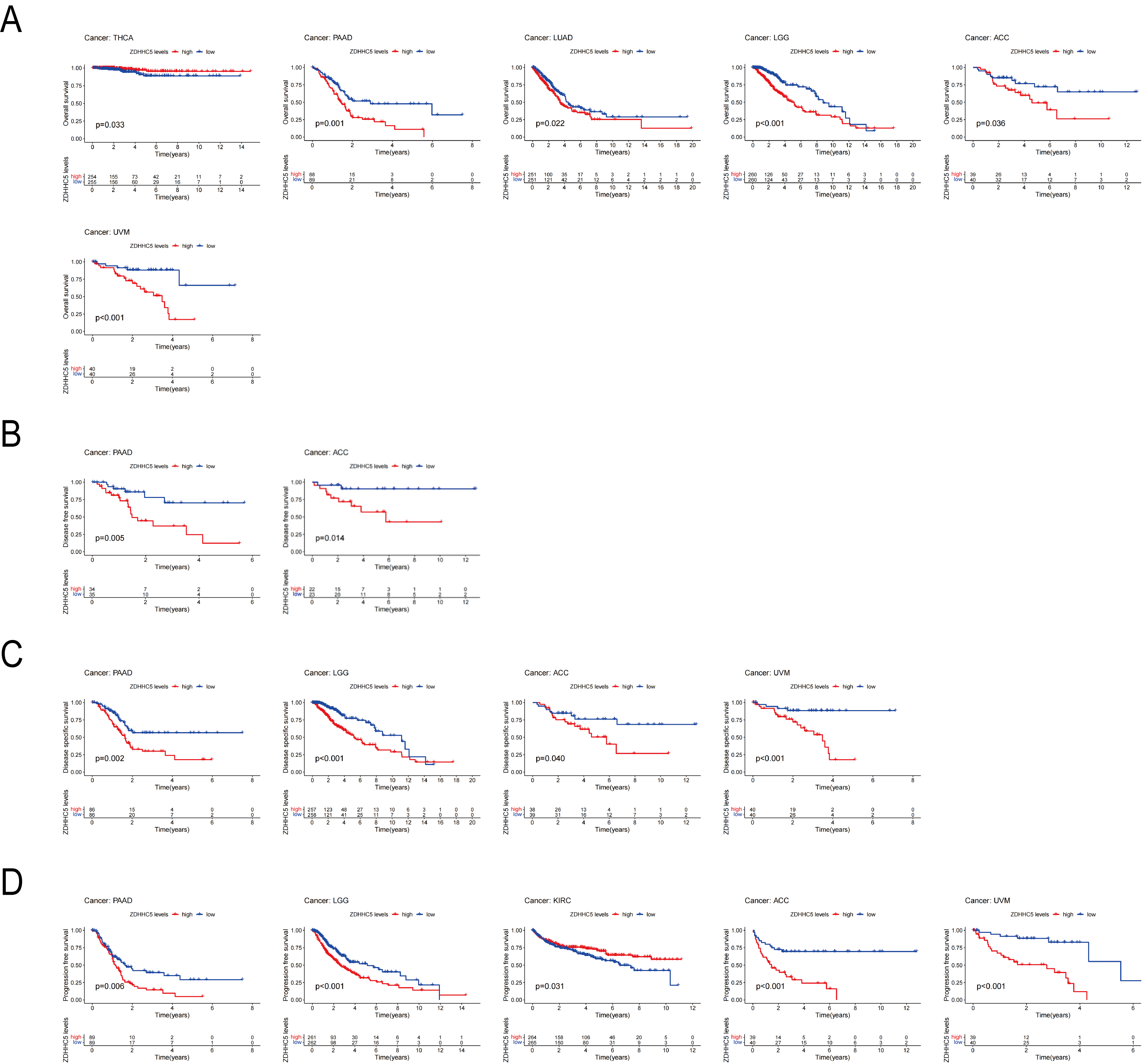

Supplement: Supplementary file 4 [file Image4.tif]

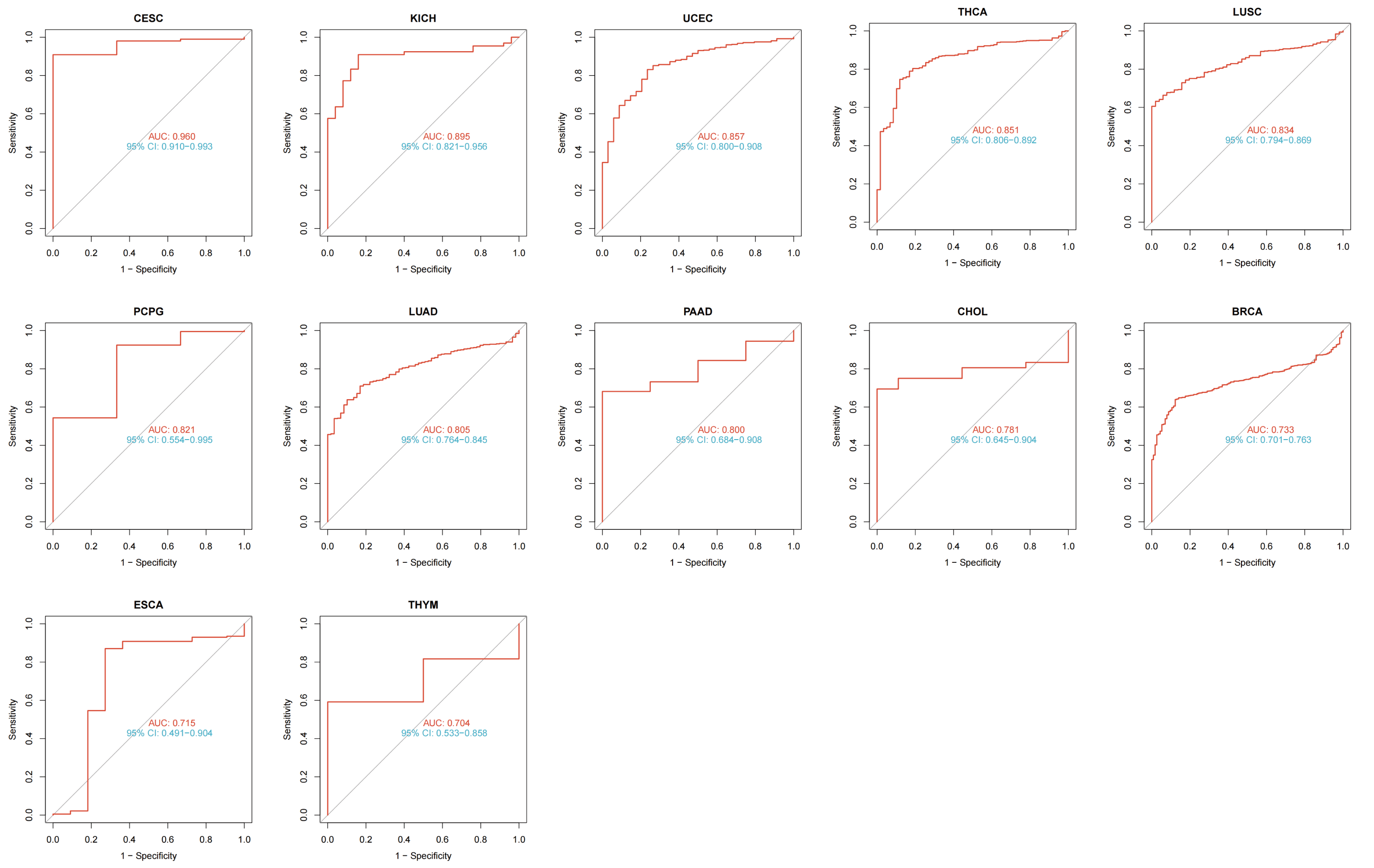

Supplement: Supplementary file 5 [file Image5.tif]

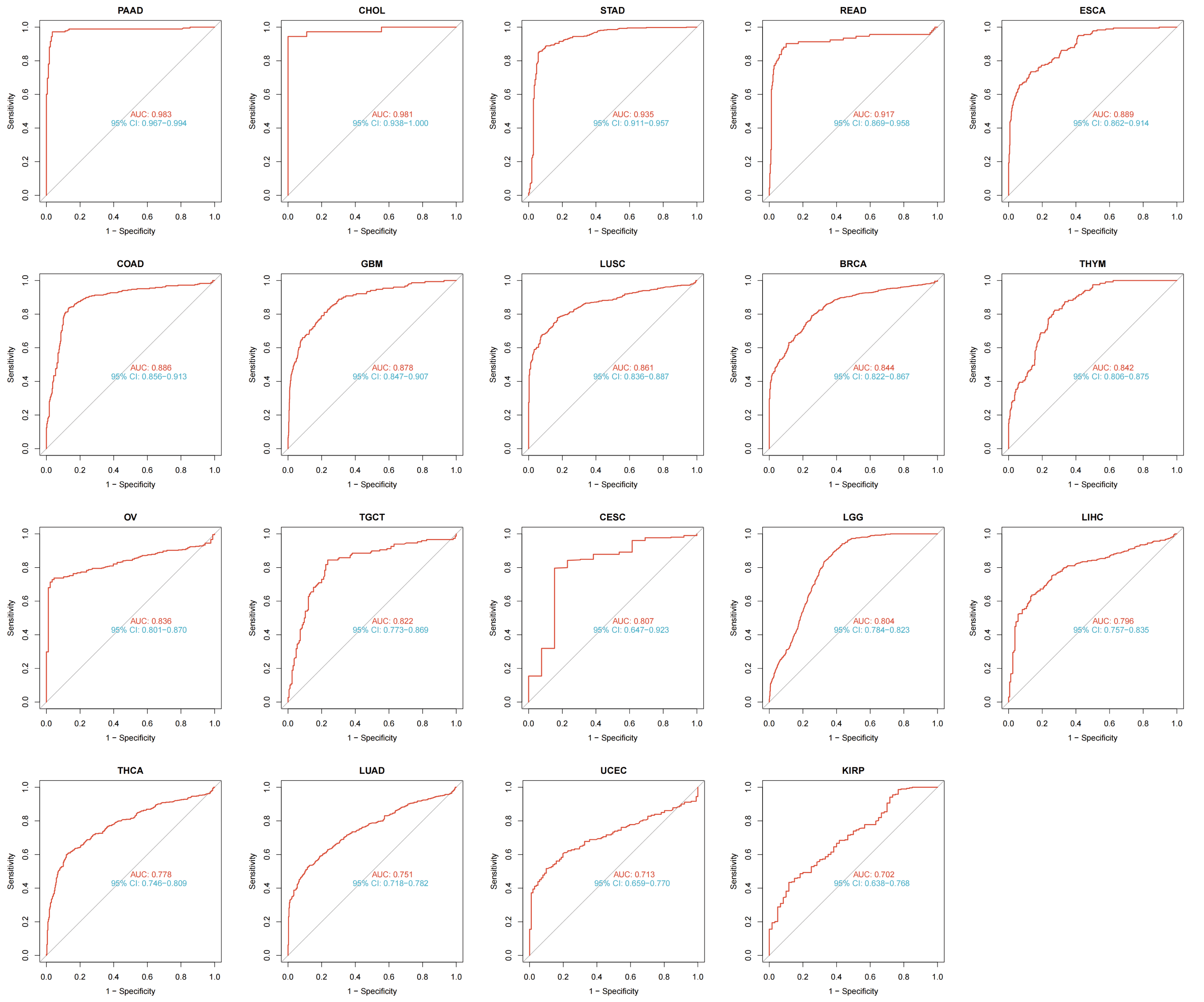

Supplement: Supplementary file 6 [file Image6.tif]

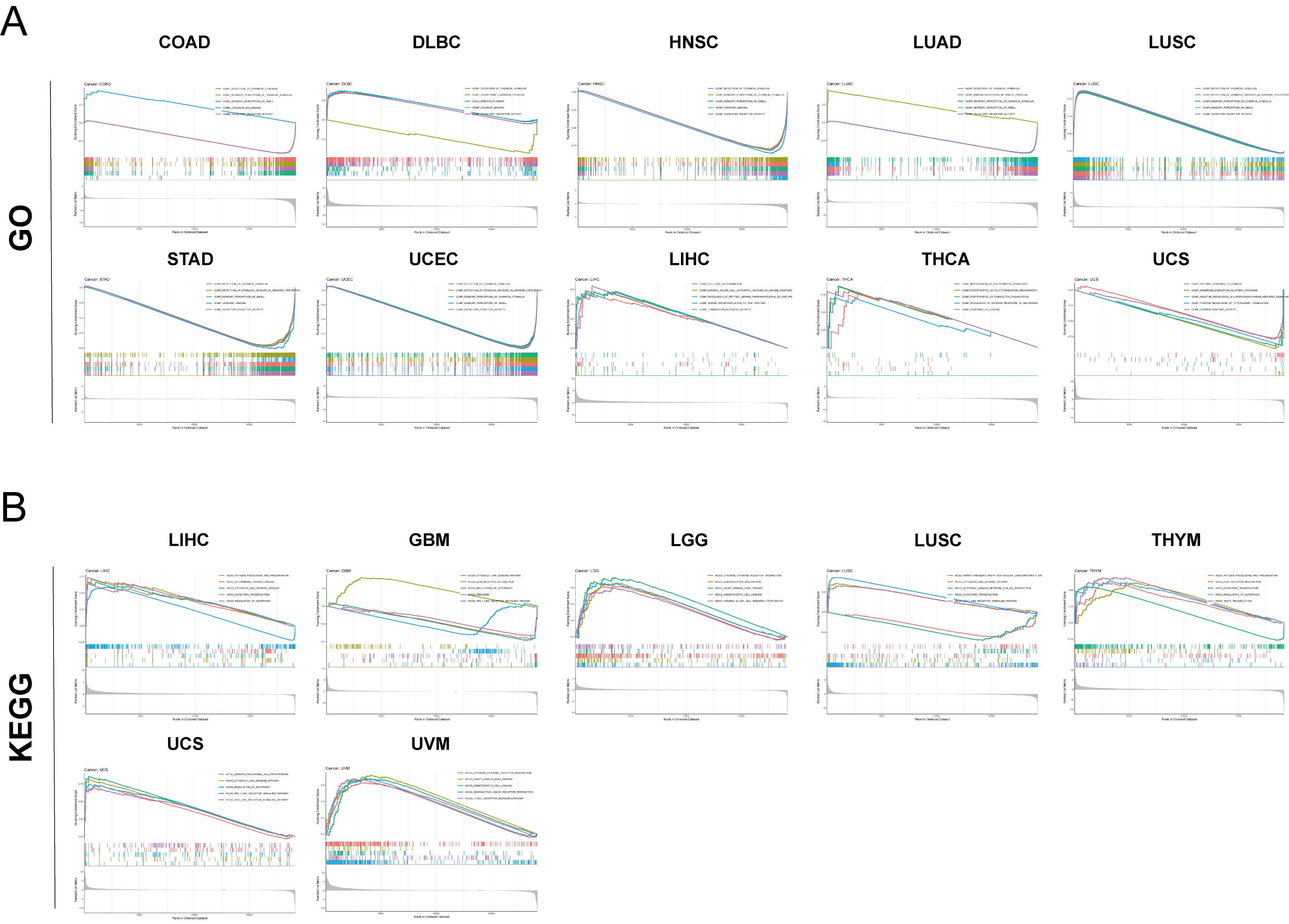

Supplement: Supplementary file 7 [file Image7.tif]

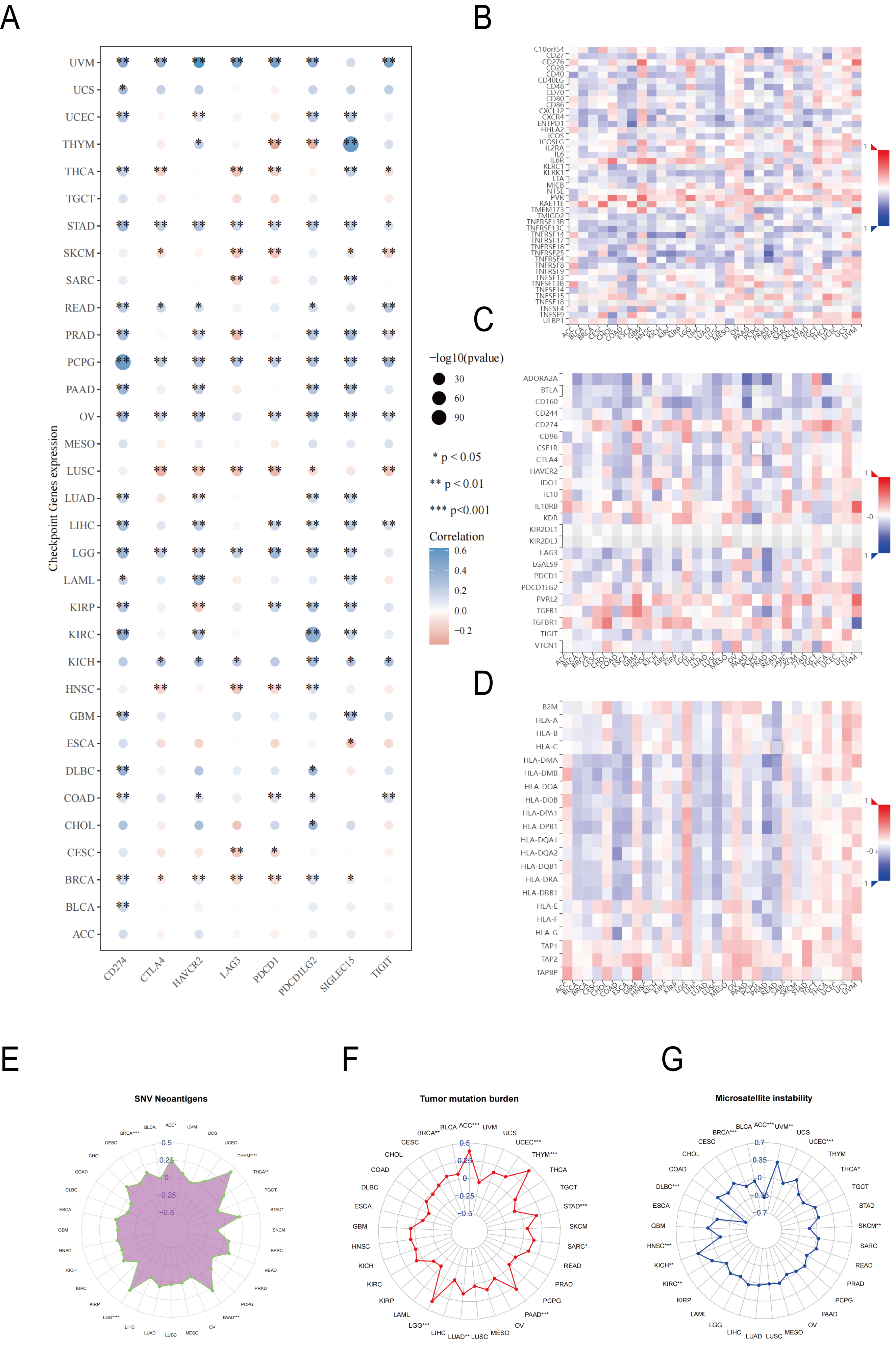

Supplement: Supplementary file 8 [file Image8.tif]

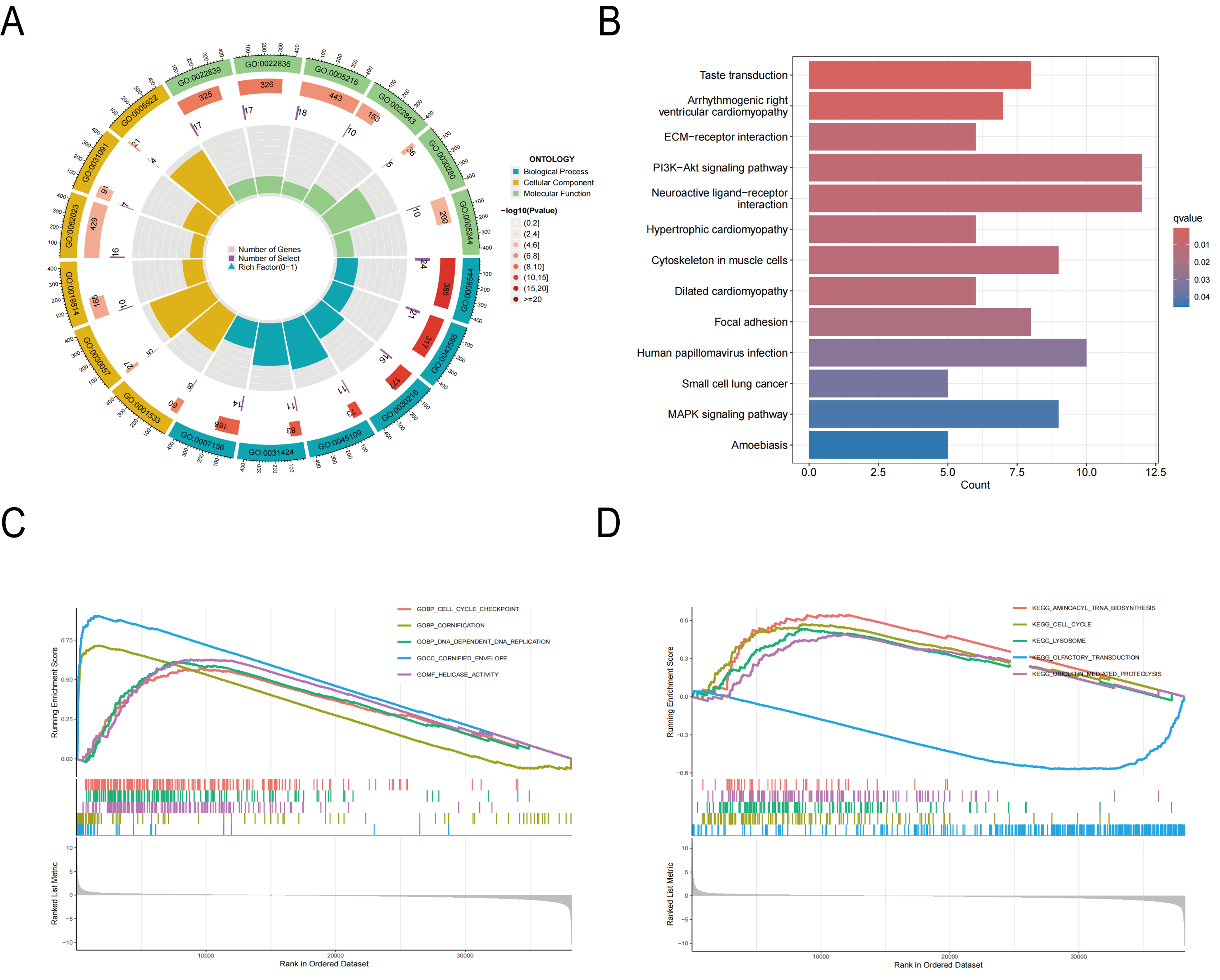

Supplement: Supplementary file 9 [file Image9.tif]

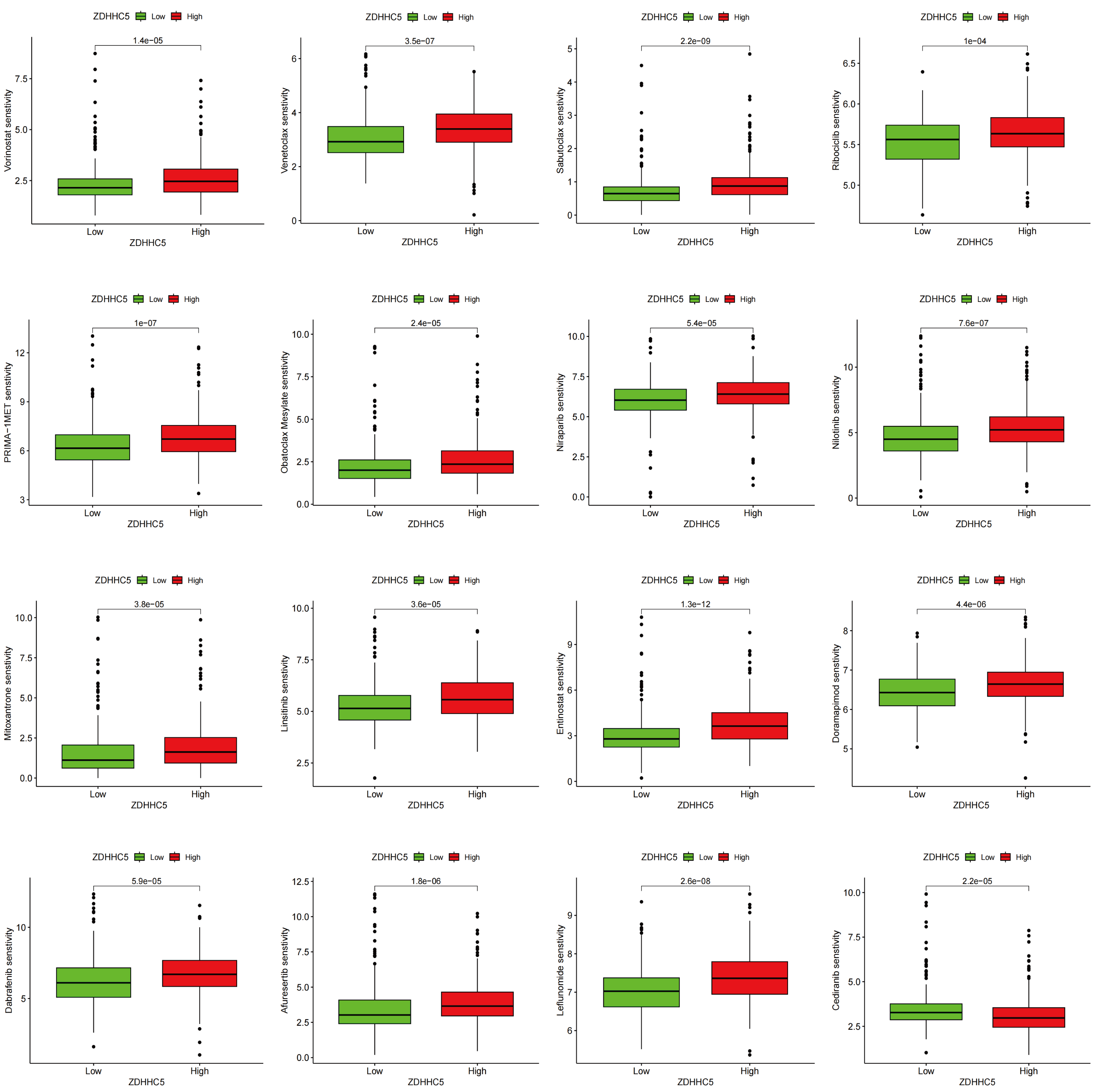

Supplement: Supplementary file 10 [file Image10.tif]

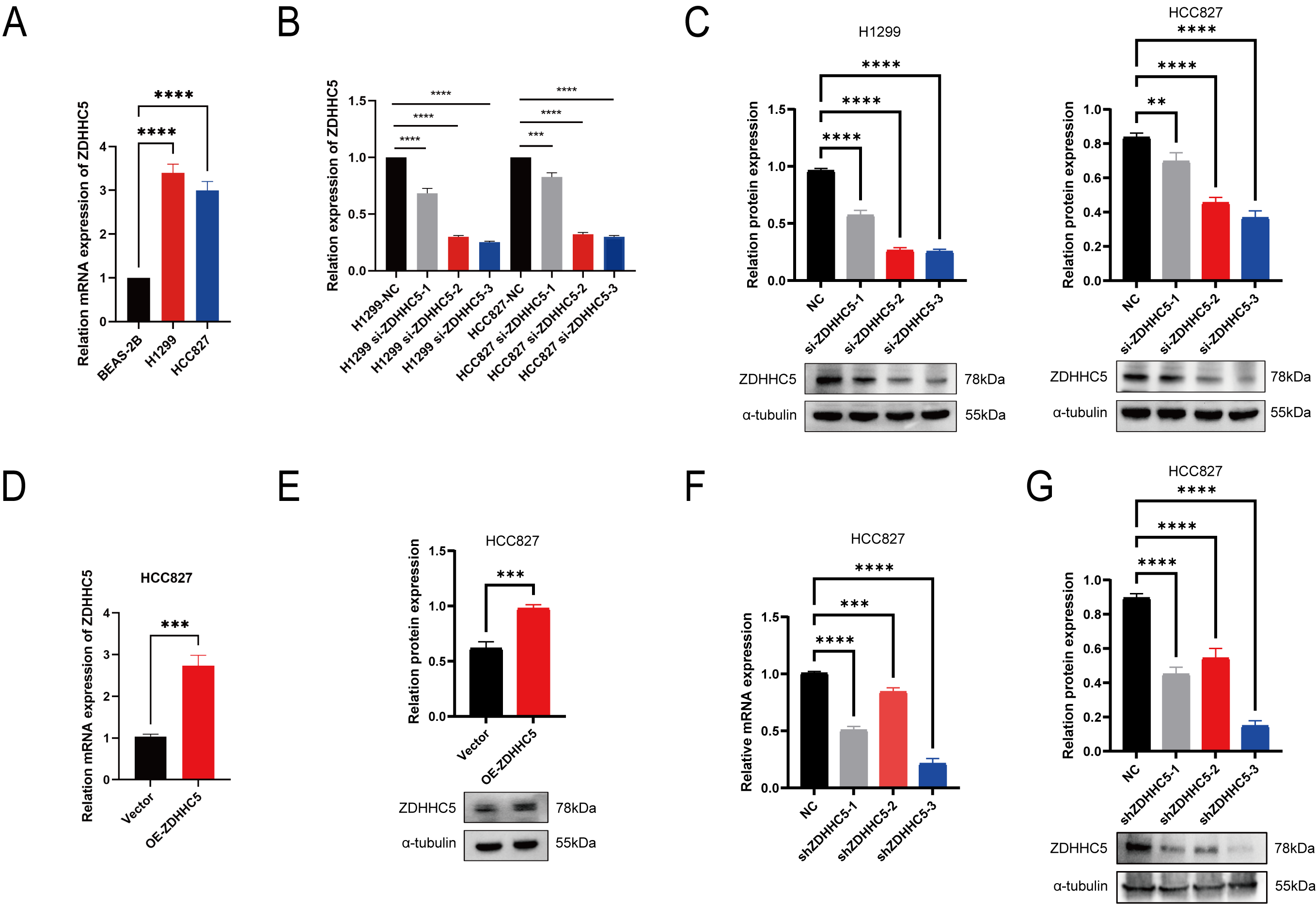

Supplement: Supplementary file 12 [file Image12.tif]
